# Examining Bald Eagle Contaminant Exposure and Reproductive Risk Above and Below Dams on Great Lakes Tributaries

**DOI:** 10.1007/s00244-024-01090-w

**Published:** 2024-10-17

**Authors:** Carly Jasmine Eakin, Lisa Williams, Jeremy Moore, Mandy Annis, David Best, Sarah Warner, William Bowerman, Latice Fuentes, Kendall Simon, Brandon Armstrong

**Affiliations:** 1 US Fish and Wildlife Service, Anchorage, Alaska USA; 2 US Fish and Wildlife Service, East Lansing, MI USA; 3 Department of Environmental Science and Technology, University of Maryland, College Park, MD USA; 4 Michigan Department of Environment, Great Lakes, and Energy, Lansing, MI USA; 5 US Fish and Wildlife Service, Boise, ID USA; 6 US Fish and Wildlife Service, East Lansing, Michigan USA; 7 US Fish and Wildlife Service, Madison, WI USA; 8 US Fish and Wildlife Service, Concord, NH USA; 9 Cincinnati, OH USA

## Abstract

Removing lowermost dams can reestablish fish passage on Great Lakes tributaries. This can increase the transfer of contaminants from anadromous fish to piscivorous wildlife upstream; however, concentrations of bioaccumulative contaminants in Great Lakes fish have decreased over the last several decades. We analyzed concentrations of PCBs and the toxic equivalence (TEQs) calculated from PCBs, DDTs, other organochlorine pesticides, and PBDEs in the plasma of bald eagle nestlings above and below lowermost dams on five river systems in Michigan from 1999 to 2013. We examined relationships between contaminants and metrics of reproductive success from 1997 to 2018, including the effects of year and location relative to the lowermost dam. Σ_20_PCB and *p,p’*-DDE were important in characterizing differences in contaminant mixtures above and below dams. Concentrations of contaminants were generally greater below dams than above. There were generally greater nest success and more nestlings per nest below dams, but nest location explained little variability (*R*^2^ values = 0.03–0.15). Neither Σ_20_PCB nor *p,p’*-DDE was a significant predictor of 5-year productivity means by river reach despite concentrations exceeding previously established effects thresholds for healthy bald eagle populations in the Great Lakes (≥ 1 nestling/nest). Our study indicates that dams may continue to reduce the upstream movement of contaminants to bald eagles, but at the measured concentrations, contaminants did not impair productivity and reproductive success as indicated by nestlings per nest. Additional information about population dynamics could clarify population-level effects of contaminants on bald eagles and to what degree these populations are self-sustaining throughout the Great Lakes.

**Supplementary Information:**

The online version contains supplementary material available at 10.1007/s00244-024-01090-w.

Restoring river connectivity by removing dams or providing for fish passage from below to above dams has received increased attention because of environmental requirements set by the Federal Energy Regulatory Commission (FERC) for relicensing hydropower facilities in the USA, hazards associated with failing facilities, and the implementation of concepts such as ecosystem-based management (Bednarek 2001). The U.S.–Canada Great Lakes Water Quality Agreement designated geographic Areas of Concern (AOC) within the Great Lakes basin with significant beneficial use impairments (BUI), which are changes to the chemical, physical, or biological integrity of the area that are sufficient to cause significant biological degradation. The Michigan Department of Environment, Great Lakes, and Energy (EGLE, formerly the Department of Environmental Quality) has identified dam removals in some of the AOCs as priority actions to assist in the delisting of “Degradation of Fish and Wildlife Populations” and “Loss of Fish and Wildlife Habitat” BUIs.

Despite the potential benefits associated with restoring river connectivity, removal of lowermost dams in Great Lakes tributaries raises multiple concerns: invasive species, disease, and contaminant-laden fish including introduced salmonids. These contaminated fish can move upstream from the Great Lakes to less contaminated areas, with ecological harm to the above-dam river ecosystem potentially outweighing benefits from dam removal (e.g., Freeman et al. 2002; Burkett et al. 2012). Exposure of fish and wildlife to legacy contaminants, including 1,1’-(dichloroethenylidene)bis(4-chlorobenzene) (*p,p’*-DDE), polychlorinated biphenyls (PCBs), and dioxin-like compounds including dioxin-like PCB congeners, has led to tumors, deformities, low reproductive rates, and ultimately population declines in AOCs and throughout the Great Lakes. For example, levels of PCBs and the toxic equivalence (TEQ) of dioxin-like compounds found in fish species were associated with adverse effects in fish-eating waterbirds in the Saginaw River and Bay AOC in Lake Huron (Tillitt et al. 1991; Yamashita et al. 1993; Ludwig et al. 1993). TEQ values are calculated using toxic equivalency factors (TEFs), which indicate the toxicity of a compound relative to 2,3,7,8-tetrachlorodibenzo-*p*-dioxin (TCDD) (Van den Berg et al. 1998). For each compound, the substance-specific TEF is multiplied by the concentration to calculate a substance-specific TEQ. Despite general declines in PCB levels in herring gulls (*Larus argentatus*) that eat fish from the Great Lakes, PCBs levels in herring gulls continue to pose a threat to herring gull populations in some areas of the Great Lakes (Brady et al. 2024).

Transfer of contaminants from the Great Lakes into connecting tributaries by migrating fish has been shown to increase risk to resident fish (Lewis and Makarewicz 1988; Scrudato and McDowell 1989; Janetski et al. 2012; Gay 2022) and piscivores upstream (Giesy et al. 1994; Giesy et al. 1995). River connectivity projects in the Great Lakes region may pose similar ecological risks by allowing the movement of Great Lakes fish with elevated contaminant levels to areas previously inaccessible due to barriers. The ecological impacts of such connectivity projects have not been well studied in recent years. Giesy et al. (1994), Giesy et al. (1995)) determined the concentrations of PCBs and TEQs in fish captured below lowermost dams on three rivers in Michigan presented a significant hazard to bald eagles (*Haliaeetus leucocephalus*) and mink (*Neovison vison*), while fish above the dams did not contain contaminant levels that would adversely affect these populations.

Although migrating fish can transfer of contaminants from the Great Lakes into connecting tributaries, declines in contaminant concentrations have been documented in recent decades. PCB concentrations have declined in many Great Lakes fish species, including those in several areas that previously had high levels of legacy contaminants (e.g., AOCs) (Muttray et al. 2020; Visha et al. 2018; Bohr and Zbytowski 2009; Madenjian et al. 2009; Jude et al. 2010). Similarly, other organochlorine (OC) pesticides such as total 1,1’-(2,2,2-trichloroethane-1,1-diyl)bis(4-chlorobenzene) (DDT), dieldrin, *cis-*chlordane, oxychlordane, and *cis-*nonachlor have declined in fish in Lake Michigan and Lake Huron associated with natural attenuation and source remediation (Carlson et al. 2010; Chang et al. 2012; Zhou 2019). PCBs and OC compounds in fish-eating waterbirds from the Great Lakes have concurrently declined with contamination in fish (de Solla et al. 2016; Freeman et al. 2002). These studies suggest that remediation and the natural attenuation of legacy contaminants may have reduced the risks to fish and wildlife in the approximately 30 years that have passed since Giesy et al. (1995) assessed the risk to bald eagle from removing the lowermost dams on three rivers in Michigan.

The overall aim of this study was to provide an updated assessment of the potential impact on bald eagle reproductive success from contaminant transfer where there is potential to reestablish fish passage above lowermost dams from the Great Lakes. The contaminant levels and associated reproductive risk to bald eagles both above and below dams on Great Lakes tributaries were hypothesized to be less during our study period (1999–2013 for contaminants and 1997–2018 for associated reproductive metrics) than were previously measured. Although co-occurring contaminant mixtures as well as resource-dependent ecological variables in the Great Lakes had changed over the previous 30 years, we anticipated that published thresholds for concentrations of single compounds or groups of compounds (i.e., PCBs) in bald eagle plasma would generally continue to predict bald eagle production during our study period. To investigate these relationships, metrics of reproductive success are based on the presence and number of nestlings per occupied territory and are comparable with metrics used in the Northern States Bald Eagle Recovery Plan (USFWS 1983) and Bowerman et al. (2003), which have been used to inform management actions for bald eagles in the Great Lakes. Risks associated with dam removal were examined at five river systems in Michigan, one of which is on the Michigan–Wisconsin border. Risks were examined by comparing legacy contaminants in the plasma of bald eagle nestlings (hereafter referred to as nestling plasma) from nests where parents foraged above or below the lowermost dam that functioned as a barrier to fish passage (hereafter referred to as above or below dam). We examined contaminants including PCBs and *p,p’*-DDE as well as other OCs and, in a subset of rivers, polybrominated diphenyl ethers (PBDEs). We assessed the risk from these contaminants above and below dams based on published thresholds for effects on bald eagle productivity and also looked directly at productivity in the areas sampled.

## Methods

### Study Sites

**Fig. 1:**
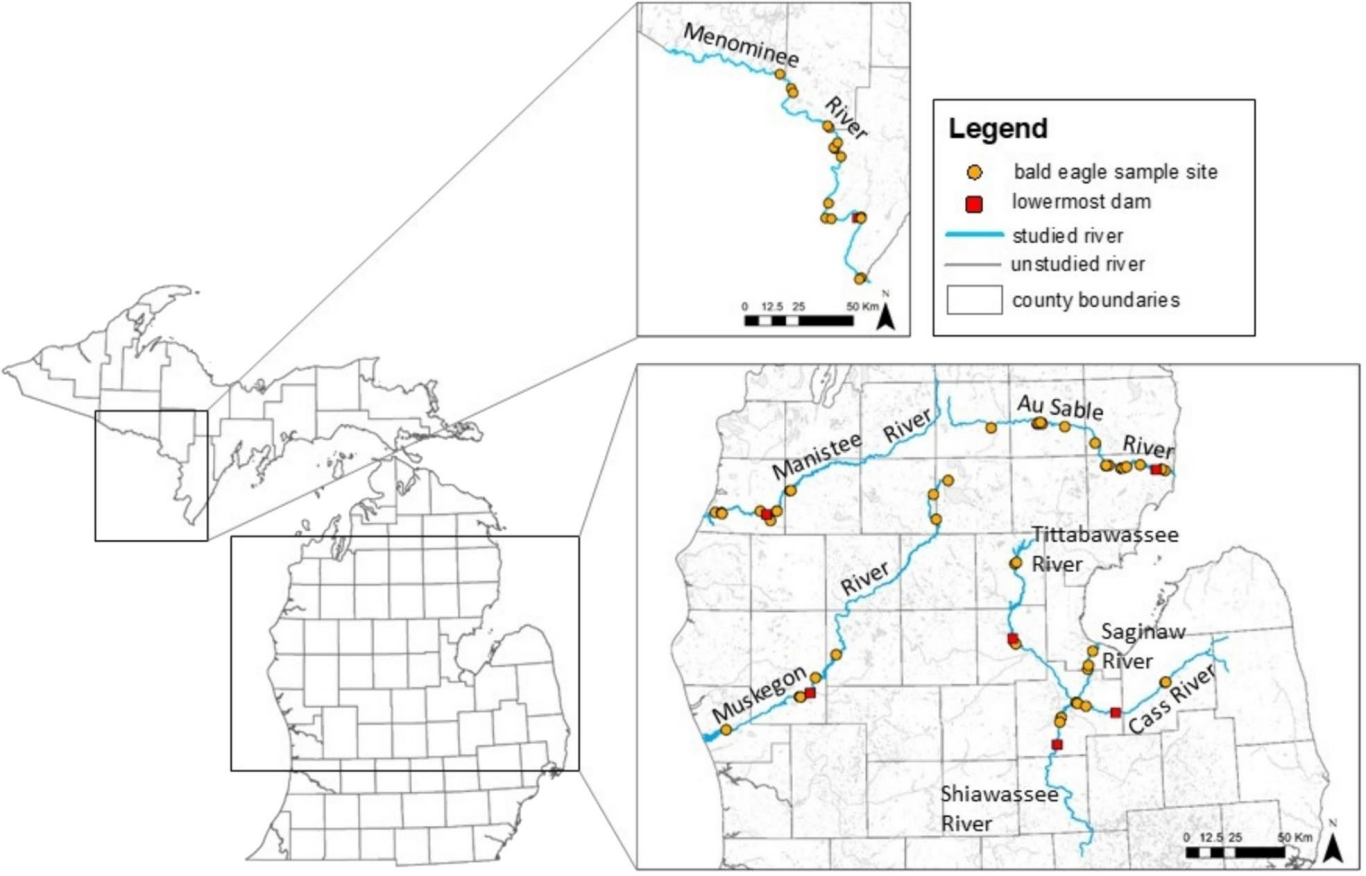
Bald eagle (*Haliaeetus leucocephalus*) nestling plasma sampling site distribution (1999–2013) and lowermost dams along the Cass, Tittabawassee, Au Sable, Manistee, and Muskegon rivers in Michigan and the Menominee River on the border between Michigan and Wisconsin. The lowermost dams on the Cass and Shiawassee rivers were replaced with rock ramps in 2014 and 2009, respectively. Circles = bald eagle sample sites; squares = lowermost dams; blue lines = studied rivers

We selected bald eagle nests in 47 occupied breeding territories, where a breeding territory is defined as an area occupied by one mated pair during the breeding season that includes one occupied nest, where breeding, nesting, brooding, or nestling rearing activities are observed (Postupalsky 1974). Based on these observations and observations that 93% of eagle prey along the Au Sable and Manistee Rivers in Michigan were fish (Bowerman 1993), we assume prey at our study sites was primarily from aquatic sources. The nests we studied represented nestlings that were fed fish from either below or above lowermost dams on the Cass, Tittabawassee, Au Sable, Manistee, and Muskegon rivers in Michigan and the Menominee River on the border between Michigan and Wisconsin. At sampled nests there was a high degree of certainty that nesting adults foraged entirely below or above a dam (Fig. 1), determined either by direct observation of feeding behaviors or given typical observed feeding ranges. The five river systems we studied were the Saginaw, Au Sable, Manistee, Muskegon, and Menominee. The lowermost reaches of the Saginaw River system and the Au Sable River have unimpeded connectivity with Lake Huron, and the lowermost reaches of the Manistee, Muskegon, and Menominee rivers have unimpeded connectivity with Lake Michigan. The Cass, Tittabawassee, and Shiawassee rivers are tributaries of the Saginaw River, and the lowermost reaches of these rivers provide the below dam data for the Saginaw River system (Fig. 1). Two territories on the Pine and Little Manistee rivers, which are on tributaries of the Manistee River below dams and within 2 and 7 km of the Manistee, were considered below dam territories for the Manistee. This study excluded samples from river reaches above the lowermost dams where significant local sources of contamination was suspected or verified by the coauthors. All samples were collected 4–88 km from a Great Lake and at least 1640 m from a lowermost dam. The lower approximately 5 km of the Menominee River, 13 km of the Muskegon River, and 32 km of the Saginaw River are within an AOC, although the Lower Menominee River AOC was delisted in 2020 (Fig. 1). The Tittabawassee River, which flows into the Saginaw River, is also a Superfund Alternative Site (i.e., a Superfund Site that needs long-term clean up that can be achieved without being listed on the National Priorities List, as determined by the U.S. Environmental Protection Agency) and the focus of a Natural Resource Damage Assessment under the Comprehensive Environmental Response, Compensation, and Liability Act. These programs support and direct contaminant remediation and restoration of natural resources associated with the Tittabawassee River.

### Nestling Blood Collection and Laboratory Analysis

This study used 132 archived plasma samples from bald eagle nestlings at 5–9 weeks old, collected 1999–2013 across 47 breeding territories. All samples (1999–2013) were collected as part of the long-term Michigan eagle sentinel project headed by the Surface Water Quality Division of EGLE except for three samples collected (1999–2013) on Blueberry Island near the mouth of the Menominee River, which were collected by the Wisconsin Department of Natural Resources (WIDNR; Table 1). Territories were sampled 1–13 years (median = 2; only 1 territory was sampled > 7 years).

**Table 1:** Analytical datasets with years of sampling, number of bald eagle (*Haliaeetus leucocephalus*) nestling plasma samples (n), sampled territories, and analytes examined in above and below lowermost dams on five river systems in Michigan, one of which is on the Michigan–Wisconsin border, 1999–2013

Laboratory	Years	n	Territory	Analytes			
				PCBs	PBDEs	DDTs	Other
GERG	2009–2013	24	17	209 PCBs (including coeluted congener sets): 4/10, 5/**8**, 7/9, 16/32, **18**/30, 20/21/33, 22, 23/34, 24/27, **28**/31, 41/68, 43/**52**, 47/48/65/75, 60/64/69, **66**/80, 70/76, 71/72, 82/107, 83/112, 85/120, 86/97/125, 87/115, 89/90/**101**, 95/121, 98/102, 106/**118**, 111/116/117, 131/142/165, **138**/158/160, 139/140/149, 146/161, **153**/168, 163/164, **170**/190, 172/191, 174/181, **180**/193, 182/**187**, 196/203, 200/201	40 congeners (1, 2, 3, 7, 8/11, 10, 12, 13, 15, 17, 25, **28**, 30, 32, 33, 35, 37, **47**, **49**, **66**, 71, 75, 77, **85**, **99**, **100**, 116, 118, 119, 126, **138**, **153**, **154**, 155, 166, 181, **183**, 190, **209**)	*o,p’-*DDE; *p,p’-*DDE; *o,p’-*DDD, *p,p’-*DDD; *o,p’-*DDT, *p,p’-*DDT	1,2,3,4-tetrachlorobenzene, 1,2,4,5-tetrachlorobenzene, aldrin, *alpha*-BHC, *alpha*-chlordane, *beta*-BHC, chlorpyrifos, *cis-*nonachlor, *delta*-BHC, dieldrin, endosulfan II, endrin, *gamma*-BHC, *gamma*-chlordane, HCB, heptachlor, heptachlor epoxide, mirex, oxychlordane, pentachloroanisole, toxaphene, *trans-*nonachlor
CIET	1999–2008	105	42	Σ_20_PCB = sum of 20 congeners (**8**, **18**, **28**, **44**, **52**, **66**, **101**, **105**, **110**, **118**, **128**, **138**, **153**, **156**, **170**, **180**, **187**, **195**, **206**, **209**) with ½ Σ_20_PCB LOD-substituted for non-detects	NA	*p,p′‐*DDE	NA
WSLH	2002	1	1	113 congeners (individual and coeluted congener sets: 3, 4/10, 6, 7/9, **8**/5, 15/17, 16/32, **18**, 19, 22, 24/27, 25, 26, **28**/31, 33, 37/42, 40, 41/71/64, **44**, 45, 46, 47/48, 49, 51, **52**, 53, 56/60, 63, **66**, 70/76, 74, 77/**110**, 82, 83, 85, 87, 89, 91, 92/84, 95, 97, 99, **101**, **118**, 123/149, **128**, 132/**153**/**105**, 135/144, 136, 137/176, 141, 146, 151, 158, 163/**138**, 167, **170**/190, 172, 174, 177, 178, **180**, 183, 185, **187**/182, 193, 194, 198, 199, 201, 202/171, 203/196, **206**, 207, 208/**195**)	NA	*p,p’*-DDE	NA
WSLH	2011–2013	2	1	72 congeners (individual and coeluted congener sets: 4/10, 6, 7/9, **8**, 15/17, 16/32, **18**, 19, 22, 25, 26, 27, **28**/31, 33, 37/42, 40, 41/71/64, **44**, 45, 46, 47/48, 49, **52**, 53, 56/60, 63, **66**, 70, 74, 77/**110**, 82, 83, 84, 85, 87, 89, 91, 92, 95, 97, 99, **101**, **118**, **128**, 130, 132/**153**/**105**, 135/144, 141, 146, 149, 151, **156**, 158, 163/**138**, 167, **170**/190, 172, 174, 177, 178, **180**, 183, 185, **187**, 193, 194, 199, 201, 202/171, 203/196, **206**, 208/**195**	17 congeners (**28**, **47**, **49**, **66**, **85**, **99**, **100**, **138**, **153**, **154**, 156, **183**, 197, 196, 207, 206, **209**)	*p,p’*-DDE; *p,p’*-DDD; *p,p’*-DDT	*cis-*chlordane, *cis-*nonachlor, dieldrin, *trans-*chlordane, *trans-*nonachlor

GERG = Geochemical and Environmental Research Group, CIET = Clemson Institute of Environmental Toxicology, WSLH = Wisconsin State Lab of Hygiene; bold PCB congeners indicate those used to calculate Σ_20_PCB; bold PBDE congeners indicate those used to calculate Σ_12_PBDE

Approximately 10 mL of blood was collected from the brachial vein for each sample and separated by centrifuge to extract plasma for analysis. Clemson Institute of Environmental Toxicology (CIET; Pendleton, SC) and Geochemical and Environmental Research Group (GERG; College Station, TX) quantified contaminants in 105 and 24 samples collected 1999–2008 and 2009–2013, respectively. Three additional samples from Blueberry Island (2002, 2011, and 2013) were analyzed by the Wisconsin State Lab of Hygiene (WSLH; Madison, WI; Table 1). Methods varied among laboratories (Online Resource 1 in supplemental data), but all used capillary gas chromatography with electron capture detection. All results were expressed on wet weight basis, and all laboratories used good laboratory practices for quality control. Units provided by laboratories were converted to the approximate concentration-by-mass equivalents (e.g., ppm and µg/L to ng/g) for statistical analyses.

Analyte concentrations reported by GERG were adjusted based on results from laboratory quality control checks. PCB-126, PCB-206, and co-eluting PCBs-43/52 concentrations reported by GERG in 2012 were reduced by the concentration detected in the blanks (0.033, 0.019, and 0.018 ng/g ww, respectively). Concentrations in a standard reference material (Lake Michigan fish tissue) indicated that 12 samples potentially had low detections of *p,p’*-DDE and mirex (30.8 and 36.8% recovery of *p,p’*-DDE, 64.5% recovery mirex), but because there were no recovery anomalies in spiked samples, values for these analyte concentrations were not adjusted.

To standardize summed data for PCBs and PBDEs among laboratories to the extent possible, similar subsets of PCB congeners and PBDE congeners were used to calculate summed PCBs (Σ_20_PCB) and summed PBDEs (Σ_12_PBDE) across samples. The 20 PCB congeners examined by CIET (Table 1) were used to calculate Σ_20_PCB across all samples except for those analyzed by WSLH, which did not include PCB-209 in 2002, 2011, or 2013 and PCB-156 in 2002. The relative concentrations of these congeners in data analyzed by GERG in this study and in Michigan bald eagle nestling plasma reported by Wierda et al. (2010) show that excluding these congeners from the summation of the 20 PCB congeners would reduce Σ_20_PCB by 0.5–5.5%. Σ_20_PCB for 2002, 2013, and 2011 WSLH data and all GERG data included congeners that coeluted with target congeners (Table 1). Σ_20_PCB were representative of Total PCBs based on the samples analyzed by GERG, which reported results in Total PCBs and by congener. For those samples, Σ_20_PCB strongly positively correlated with Total PCBs (Pearson’s *r* = 0.999) and contributed 51.9–84.3% (mean = 60.8%, median = 59.3%) of Total PCBs. The 12 PBDEs in common between GERG and WSLH (Table 1) were used to calculate Σ_12_PBDE.

For samples analyzed by GERG, TEQs were calculated for mono- and non-*ortho*-PCB congeners (i.e., dioxin-like PCBs (DL-PCBs)) contributing dioxin-like toxicity similar to that of 2,3,7,8-tetrachlorodibenzo-*p*-dioxin (TCDD; PCB-77, -81, -105, -114, -118, -123, -126, -156, -157, -167, -169, and -189) using toxic equivalency factors (TEFs) for birds from Van den Berg et al. 1998. We refer to data from all laboratories were merged for analysis, and two data sets were created to compare the effect of substituting values ≤ Lowest Level of Detection (LOD) with ½ LOD or zero. Analyses were initially conducted with both data sets, where applicable, and produced similar results except for when comparing the relative composition of contaminant mixtures (using the 29 contaminants examined by GERG; Table 1). Because of this overall similarity in results and because substitution with ½ LOD provides a more protective approach than substituting with zero, only results for the data set with ½ LOD substitution are presented here for all analyses except the composition of contaminant mixtures. Σ_20_PCB concentrations from CIET had already incorporated ½ LOD substitution by congener and to maintain continuity among pooled data, only ½ LOD substitution was used to calculate Σ_20_PCB.

### Hazard Assessment

Concentrations of contaminants detected in ≥ 25% of samples analyzed for each respective analyte were compared against previously published effect levels to estimate the severity of hazard posed by each contaminant within each river system and by location above or below dam. Hereafter, we refer to the contaminant concentrations associated with effect endpoints as toxicity values (TVs). Previously published effect levels for bald eagle nestling plasma were used to estimate TVs when available as detailed in the Results. When information about the effects of bald eagle nestling plasma concentrations on productivity was sparse for a given contaminant, effects of concentrations in plasma of adult bald eagles and other birds and effect endpoints other than productivity were considered for context. A tenfold safety factor was applied to estimates of adult effect thresholds to estimate TVs for nestlings (Elliott and Bishop 2011).

Where TVs directly applicable to bald eagles nesting plasma were available, a hazard quotient (HQ) was calculated by dividing the detected concentration of a contaminant in bald eagle nestling plasma by an approximate TV associated with healthy productivity (1.0 young/occupied nest; Grier et al. 1983) or population stability (0.7 young/occupied nest; Sprunt et al. 1973). A HQ > 1 represents a concentration of a contaminant that is above the threshold at which risk of a negative response would be expected. Only concentrations > LOD were used in hazard assessments except for Σ_20_PCB and DL-PCBs, which were calculated with ½ LOD substitution for congeners detected at ≤ LOD.

### Statistical Analysis

#### Reproductive Success

Data on reproductive success were collected as part of the long-term Michigan eagle sentinel project using paired aerial surveys, with the first flight during nesting and brood-rearing season to detect behavior indicating an occupied nest prior to hatching (terminology following Postupalsky 1974) and a second flight after hatching but before fledging to determine nest success and the number of nestlings hatched. For our study, we used the subset of reproductive data from nesting territories that also had contaminant data to calculate reproductive success. We selected territories where nestlings were primarily fed aquatic food sources from either above or below lowermost dams as verified during field work collecting contaminant data. Reproductive data were used from all years at these territories, not just years concurrent with contaminant data collection.

All statistical analyses were performed with R version 3.5.0 (2018). We used two sets of generalized linear mixed models (GLMM, package ‘nlme’; Pinheiro et al. 2018) to estimate the effects of nest location relative to the lowermost dam (above vs. below; hereafter referred to as Location), year of observation (Year), *p,p’*-DDE, and Σ_20_PCB on nest success and the number of nestlings per nest. We applied mixed models to account for a lack of independence among observations (Zuur et al. 2009) from the same year or river system. We modeled both nest success (0, 1) and nestlings per nest because nest success is the more accurate measure when observed from an airplane while the number of nestlings provides more detail about the reproductive contribution to the population. Throughout all statistical analyses, Year was centered on zero to reduce correlation between slope and intercept, and contaminant concentrations were natural log-transformed.

First, we modeled nest success and nestlings explained by Location and Year using binomial and Gaussian distributions, respectively (‘lme4’; Bates et al. 2015). Because reproductive success observations were available at many territories for more years than there were for contaminant observations, we were able to examine the effects of Location and Year at a finer resolution and over a longer time than if only considering the years in which contaminant data were collected (1997–2018 and *n* = 784 versus 1999–2013 and *n* = 129). Model fit was determined using pseudo-* R*^2^, *a la* Dobson 2002 for models of nestlings per nest and the Hosmer and Lemeshow goodness of fit test for probability of nest success.

Second, we modeled five-year means of nest success and of nestlings per nest explained by Location, Year, *p,p’*-DDE, and Σ_20_PCB. We calculated these means by territory using the year of a sample, the two years previous, and the two years following the year of a sample, which included years of nest success (i.e., ≥ 1 nestling produced) as well as years of nest failure (i.e., 0 nestlings). Natural log-transformed values for Σ_20_PCB and *p,p’*-DDE were tested for correlation (Pearson’s *r*). If strongly correlated (|*r*|≥ 0.7), they were examined for their contributions within separate models.

We selected GLMM model structure by fitting a full fixed effects model (first model set: Location*Year; second model set: Location*Year*contaminant; parameters that do not vary among individuals or groups) using restricted maximum likelihood (REML) estimation. Then we selected random effects (none, random intercept by river system (River), random slope and intercept by Year and River; parameters that randomly vary among individuals or groups) using the likelihood ratio test and selected fixed effects using maximum likelihood estimation. The Akaike information criterion (AIC) was used to select optimal random effects structures (Online Resource 2 in supplemental data) and a backwards stepwise selection process was used to select optimal fixed effect structures (*p* = 0.05). Final models using REML were interpreted.

We also conducted regressions for mean nestlings per occupied nests within a five-year period and by river reach that were comparable with the examination of these relationships among larger subpopulations by Bowerman et al. (2003), who used these regressions to determine threshold concentrations associated with healthy and sustainable bald eagle populations. We regressed the arithmetic mean productivity for each river reach (above and below dam reaches) within five-year increments (1999–2003, 2004–2008, 2009–2013) against the geometric means of Σ_20_PCB and *p,p’*-DDE (½ LOD data set, only). These regressions only used nests where feeding behavior had been verified and contaminant data were collected at least one year; thus, all included territories had at least one year with nest success. This bias toward successful territories may overestimate productivity in comparison with methods used by Bowerman et al. (2003) where productivity was calculated using all occupied territories within a subpopulation, including territories that were never successful within a five-year period. To examine the possible overestimate, we conducted parallel regressions of productivity using a dataset of productivity calculated from all occupied territories along river reaches, with and without contaminant measurements. Although feeding behavior was unverified in territories where contaminants were measured, we only selected territories within 1640 m of rivers and at least 1,640 m from lowermost dams. We did this to match the spatial distribution of the territories with contaminant data in hopes that these unstudied nests similarly fed nestlings aquatic food from above or below a lowermost dam.

#### *p,p’*-DDE and Σ_20_PCB

We estimated the effects of Location and Year on *p,p’*-DDE and Σ_20_PCB using GLMMs and following the same model selection process that was used for modeling *p,p’*-DDE and Σ_20_PCB. We also estimated effects of Location and Year on *p,p’*-DDE using regression equations for singly censored data (using maximum likelihood estimation; package ‘NADA’, Lee 2020), with strengths and weaknesses contrasting those of GLMMs. These models rely on an assumed distribution to handle values ≤ LOD and thus do not require substitution for individual values ≤ LOD. However, these models cannot handle random factors to account for the lack of independence among observations from the same year (Year) or river system (River). Using regression for non-substituted (i.e., censored) data, a full model estimating *p,p’*-DDE was fit with Location, Year, and a Location*Year interaction terms. Backwards stepwise variable elimination (*p* = 0.05) was used to select the final model. Σ_20_PCB was not modeled using regression for censored data because Σ_20_PCB requires ≤ LOD substitution to calculate, and thus is a composite metric which would not be appropriate for this modeling technique.

#### Composition of Contaminant Mixtures

Differences in the composition of contaminant mixtures between Locations and among Years were tested using nonparametric MANOVA (NPMANOVA, adonis2 in ‘vegan’; Oksanen et al. 2019) for a subset of samples with data for 29 contaminants (all samples analyzed by GERG, *n* = 24). One thousand permutations were run using Bray–Curtis similarity matrices with observations nested by River. Significant covariates were retained in final models (*p* = 0.05). NPMANOVA was conducted for ½ LOD- and zero-substituted datasets.

If there was a significant relationship between Location or Year and contaminants mixtures, the relative importance of each contaminant in differentiating between Locations or among Years was examined using Random Forest Analyses (RFA), a Classification and Regression Tree (CART) analysis (‘randomForest’; Liaw and Wiener 2002). Ten thousand regression trees (random forest error stabilized at ~ 2000 trees for each response variable) were built, bootstrapping with replacement and using 2/3 of the data at each iteration. Explanatory variable importance was ranked using the mean percent decrease in accuracy resulting from removal of each. Partial dependence plots (‘randomForestSRC’; Ishwaran and Kogalur 2014) were used to examine the marginal effects of predictor variables while holding all other predictors at average values (Friedman 2001; Cutler et al. 2007). Because partial dependence plots display general trends, all reported values are approximate.

#### Dioxin-Like PCBs

For samples analyzed for all PCBs (those analyzed by GERG), Total PCBs, DL-PCBs, and the ratio of DL-PCBs/Total PCBs were calculated using zero-substitution for values ≤ LOD for both total PCBs and DL-PCBs. Correlation among DL-PCBs, Total PCBs, and Σ_20_PCB was examined using Pearson’s correlation coefficient (*r*) to indicate the variability in dioxin-like toxicity contributed by PCBs. Kruskal–Wallis and Wilcoxon rank sum nonparametric tests were conducted to examine differences in DL-PCBs among Years and between Locations, respectively.

## Results and Discussion

**Fig. 2:**
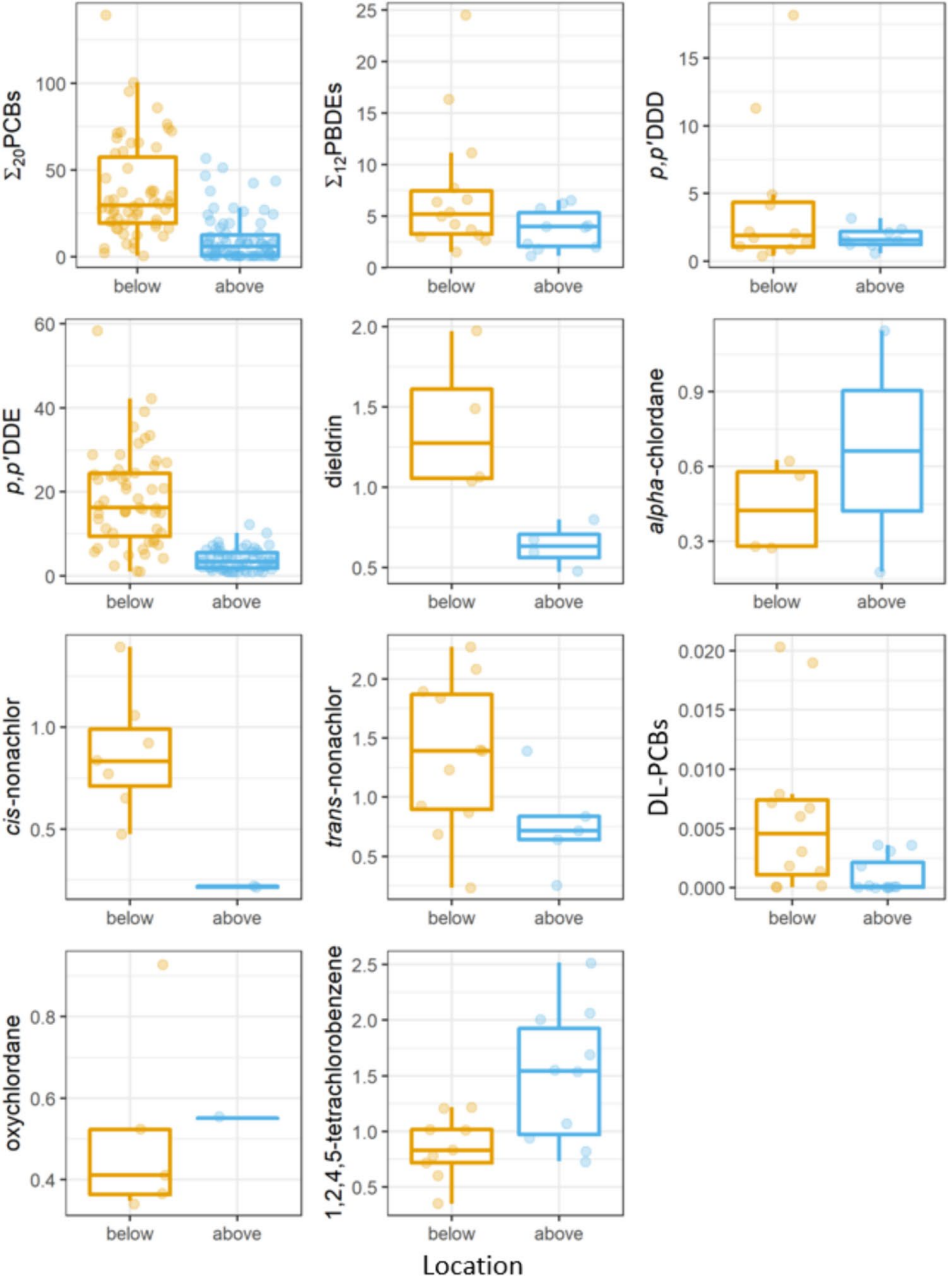
Boxplots of analytes measured at concentrations > LOD in ≥ 25% (≥ 6) of samples and DL-PCBs in bald eagle (*Haliaeetus leucocephalus*) nestling plasma in five river systems in Michigan, one of which is on the Michigan–Wisconsin border, 1999–2013. DL-PCBs are based only on PCB contributions. All concentrations are in ng/g ww. Boxplots display the 25, 50, and 75% quantiles with upper whiskers extending to the largest value and lower whiskers extending to the smallest value not further than 1.5 times the inter-quartile range (IQR). Individual measured concentrations are shown as transparent dots overlaid on boxplots and randomly distributed horizontally for ease of viewing

Ten analytes were detected in ≥ 25% (≥ 6, n varies by contaminant, Table 2 and Online Resource 3 in supplemental data) of samples, including Σ_20_PCBs, Σ_12_PBDE, seven OCs (*alpha*-chlordane, *cis-*nonachlor, dieldrin, oxychlordane, *p,p’*-DDD, *p,p’*-DDE, *trans-*nonachlor), and 1,2,4,5-tetrachlorobenzene, with > 75% detection in samples examined for Σ_20_PCBs, Σ_12_PBDE, *p,p’*-DDD, *p,p’*-DDE and 1,2,4,5-tetrachlorobenzene. The summary of concentrations of all examined analytes and DL-PCBs presented by River and Location is in Online Resource 3 in supplemental data. Contaminant concentrations were generally higher below than above dams, as was the case for Σ_20_PCB, *p,p’*-DDE, and DL-PCBs as well as seven other contaminants detected in ≥ 25% of samples (Table 2, Fig. 2). Sample size was positively correlated with the number of contaminants detected in a river system (Online Resource 4 in supplemental data); thus, a lack of detection should not necessarily be assumed to indicate the absence of a contaminant.

**Table 2:** Summary statistics by Location (above or below lowermost dams) for analytes measured at concentrations > LOD in ≥ 25% (≥ 6) of samples in bald eagle (*Haliaeetus leucocephalus*) nestling plasma in five river systems in Michigan, one of which is on the Michigan–Wisconsin border, 1999–2013

Analyte	> LOD (*n*)	*n*	Location	Median	Geo mean	Min	Max
Σ_20_PCB	50	74	Above	8.47	9.90	2.00	57.0
	57	58	Below	30.6	29.4	2.33	139
Σ_12_PBDE	10	12	Above	3.96	3.27	1.13	6.50
	14	14	Below	5.17	5.49	1.51	24.5
*p,p’*-DDD	8	12	Above	1.55	1.53	0.557	3.19
	12	14	Below	1.88	2.20	0.387	18.2
*p,p’*-DDE	59	74	Above	3.40	3.09	1.00	12.3
	55	58	Below	16.2	14.4	1.07	58.3
Dieldrin	4	12	Above	0.634	0.624	0.474	0.799
	4	14	Below	1.28	1.34	1.05	1.98
*Alpha*-chlordane	2	12	Above	0.663	0.454	0.180	1.15
	4	12	Below	0.423	0.407	0.274	0.626
*Cis-*nonachlor	2	12	Above	0.217	0.216	0.212	0.221
	7	14	Below	0.833	0.829	0.474	1.39
*Trans-*nonachlor	5	12	Above	0.717	0.672	0.255	1.39
	11	14	Below	1.39	1.16	0.236	2.27
Oxychlordane	1	12	Above	0.551	0.551	0.551	0.551
	5	12	Below	0.411	0.479	0.347	0.933
1,2,4,5-tetrachlorobenzene	10	12	Above	1.54	1.38	0.729	2.52
	9	12	Below	0.831	0.808	0.349	1.22

Concentrations in ng/g ww Min = minimum measured concentration; Max = maximum measured concentration.

### Reproductive Success

We examined the effects of Location and Year for the 784 occupied nest observations from the studied territories from 1997 to 2018, with 655 of these observations from years when contaminant data were not collected (271 below and 513 above dams). We observed 0–3 nestlings per nest (mean = 1.15, median = 1.00; below dams: mean = 1.33, median = 1; above dams: mean = 1.06, median = 1) and at least 1 nestling was detected in 70.5% of observations. Fewer nestlings per individual occupied nest were predicted above than below dams (below dam as the reference Location: *β*_Location_ = − 0.263, *p* = 0.000115) and with each passing year (*β*_Year_ = − 0.0168, *p* = 0.00193) when modeling the effects of Location and Year. However, the model accounted for only 3% of the variance in nestlings (pseudo-* R*^2^, *a la* Dobson 2002 = 0.0311). Similarly, the probability of nest success (≥ 1 nestling observed) was 41.3% less above than below dams (*β*_Location_ = − 0.532, *p* = 0.00216) and 2.7% less each passing year (*β*_Year_ = − 0.0273, *p* = 0.0388), but model fit was poor (Hosmer and Lemeshow goodness of fit test: χ^2^ = 28.387, df = 8, *p* = 0.000406).

We examined the effects of Location, Year, Σ_20_PCB, and *p,p’*-DDE on five-year means of nestlings per occupied territory and mean nest success for the 129 territory-years for which Σ_20_PCB and *p,p’*-DDE data were available (57 territory-years below dams and 72 above). Productivity averaged from 0.40 to 2.60 nestlings per nest per year for different combinations of Location and Year (overall mean = 1.42, median = 1.40; above dam mean = 1.33, median = 1.25; below dam mean = 1.54, median = 1.6) and, similarly, mean nest success over five years ranged from 40 to 100% (overall mean = 81%, median = 80%; above dam mean = 77%, median = 80%; below dam mean = 87%, median = 80%). Similar to the models not including contaminants, in these models including contaminants, significantly more nestlings per nest were predicted below dams as well as at higher Σ_20_PCB concentrations, but the models explained only approximately 6% of the variance (adj. *R*^2^_Location_ = 0.0548, adj. *R*^2^_Σ20PCB_ = 0.0566; Table 3). Greater mean nest success was also predicted below dams (full model including Σ_20_PCB). When *p,p’*-DDE was included in the full model, the combined effect of Year and *p,p’*-DDE contributed to greater mean nest success *above* dams (significant three-way interaction; Table 3). Although other non-significant parameters remained in the model, all other significant two-way and single term effects in the final model indicated lower mean nest success above dams and that, overall, each passing year was associated with greater nest success. However, both nest success models explained relatively little variance in the data (adj. *R*^2^_Location_ = 0.0917, adj. *R*^2^_*p,p’*-DDE*Location*Year_ = 0.147. Natural log-transformed Σ_20_PCB and *p,p’*-DDE were strongly correlated (Pearson’s *r* = 0.691) and thus were examined in separate models. Only fixed effects were maintained in final models.

**Table 3:** Covariates for models of the five-year means of nestlings per occupied territory and nest success associated with nests where bald eagle (*Haliaeetus leucocephalus*) nestlings were sampled for contaminants above and below lowermost dams across five river systems in Michigan, one of which is on the Michigan–Wisconsin border, during 1999–2013

Contaminant in full model	*β*_0_	*β*_x_ covariate	*β*_x_	95% CI	*F*	*p*	adj. *R*^2^
*Nestlings per occupied territory*							
*p,p’*-DDE	1.54	Location	− 0.203	− 0.351, − 0.0550	7.37	0.00757	0.0548
Σ_20_PCB	1.23	Σ_20_PCB	0.0784	0.0222, 0.135	7.62	0.00665	0.0566
*Nest success*							
*p,p’*-DDE	0.950	*p,p’*-DDE/Location/Year Location/Year Location Year	0.0292 − 0.0672 − 0.253 0.0389	0.00864, 0.0498 − 0.113, − 0.0216 − 0.453, − 0.0526 0.000345, 0.0775	4.16	0.00577 0.00417 0.0138 0.0480	0.147
Σ_20_PCB	0.869	Location	− 0.103	− 0.157, 10.0483	13.92	0.000286	0.0917

Covariates in original models included Location, Year, and Σ_20_PCB or *p,p’*-DDE. Contaminant covariates were natural log-transformed + 1. Below dam is the reference level for Location. *df* = 1, 127 for all final models except the *p,p’*-DDE model for nest success, with *df* = 7, 121. Only significant (*p* < 0.05) covariates are presented.

Overall, fewer nestlings per nest and lower nest success were predicted above than below lowermost dams, and some models also predicted fewer nestlings with each passing year and at lower Σ_20_PCB concentrations. Because these models explained little variance in nestling numbers or nest success, contaminant levels measured during the years of this study were unlikely to have a dramatic influence on those parameters. This suggests other unmeasured factors may explain more of the variability in these reproductive metrics. For example, food and other habitat conditions can have an important influence on bald eagle reproduction, especially as contaminant levels decline (reviewed in Elliott and Harris 2001/2002). Results of some studies have suggested that food availability may influence bald eagle reproductive rates more than PCBs or *p,p’*-DDE in nestling plasma when overall concentrations of contaminant mixtures are lower than they previously were in many areas of the Great Lakes (Dykstra et al. 2005, 1998; Gill and Elliott 2003).

The dramatic population growth coupled with a shift in bald eagle population dynamics throughout the Great Lakes between the late 1980s/early 1990s and 2013 may also help explain the change in relationships between any contaminant and productivity in our results. During this time, this population transitioned from being a relatively closed population with little nest turnover and few if any "floating" (i.e., non-breeding adults that are capable of breeding) adults, to a more open population with a greater nest turnover, especially near the Great Lakes (Simon 2013), and a large number of floating adults available to replace other adults (e.g., in case of adult mortality) without causing a break in nest occupancy. The availability of more floating adults also makes it possible for adults from areas with contaminant mixtures with lower toxicities (e.g., above dams) to move to an areas of contaminant mixtures with higher toxicities for breeding (e.g., below dams), thus temporarily bolstering productivity in an area with higher contaminants.

The significance of Location in these models also suggests that there is an underlying effect of position relative to a lowermost dam on nestling numbers and nest success. This aligns with other recent findings that demonstrate higher productivity near the Great Lakes despite elevated contaminant concentrations in nestling plasma compared to inland areas from 2006 to 2018 (5-year means; Bush and Bohr 2012, 2015; Bush et al. 2020). The slight reductions of nestling numbers and nest success above dams and with each successive year may reflect density dependent effects associated with increases in the Michigan bald eagle population (population increases noted in Simon et al. 2020). Reduced productivity due to density-dependent effects has also been described for bald eagles in inland areas of Wisconsin (Bowerman et al. 1995). Although pelagic prey reductions have led to reductions in nutrition available to fish-eating water birds in the Great Lakes (Hebert et al. 2008), the Great Lakes may continue to provide more abundant prey resources than are available inland. During spring migration in the Great Lakes, bird densities are greatest in areas near the shoreline (Archibald et al. 2017), potentially increasing the availability of these prey in eagle territories nearer to the Great Lakes.

**Fig. 3:**
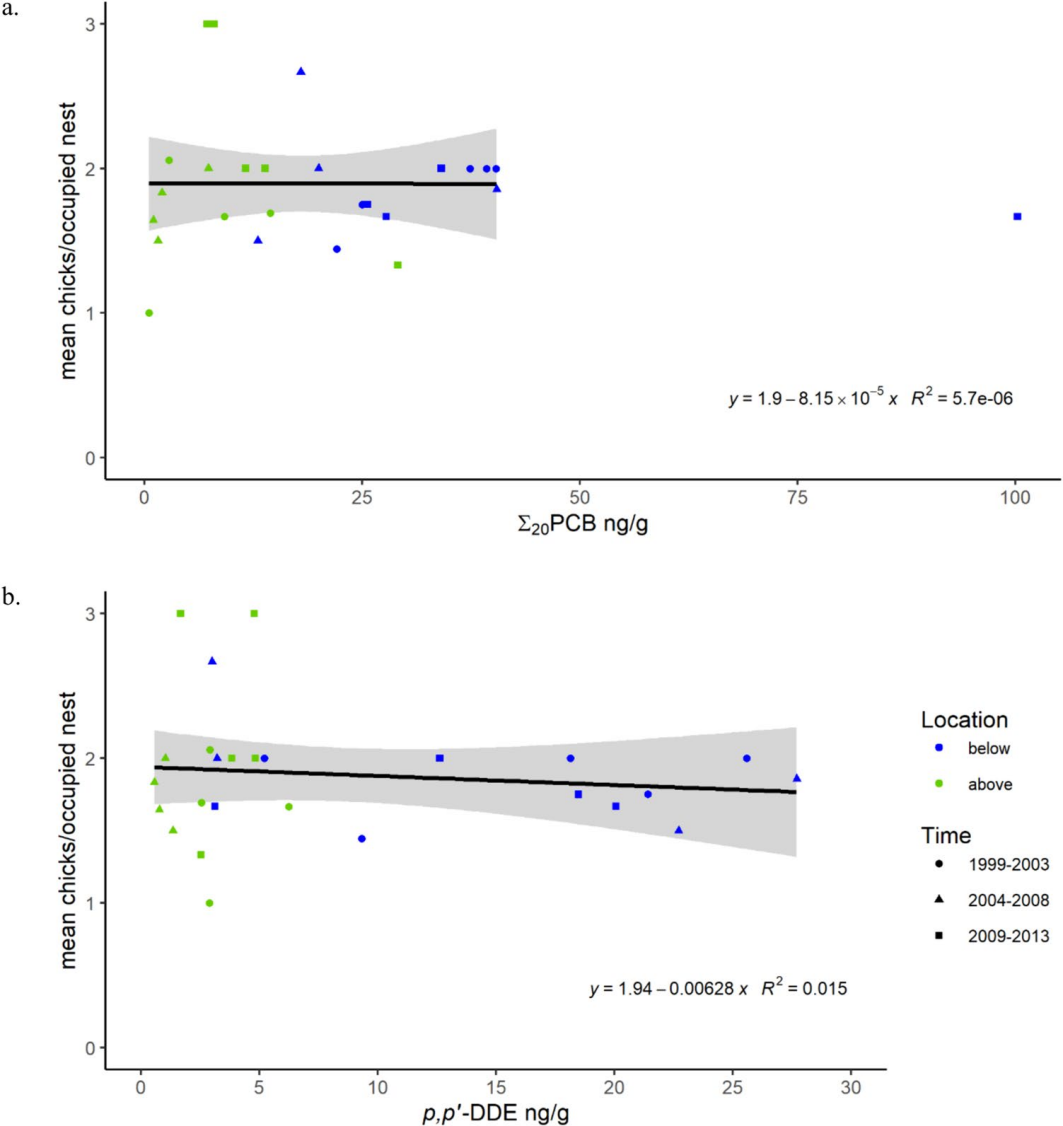
Relationship between productivity (mean nestlings/occupied nest) in five-year increments (1999–2003, 2004–2008, 2009–2013) and geometric mean concentrations of **a** *p,p’*-dichlorodiphenyldichloroethylene (*p,p’*-DDE) and **b** summed polychlorinated biphenyls (PCBs) in plasma of nestling bald eagles (*Haliaeetus leucocephalus*) above and below lowermost dams along five river systems in Michigan, one of which is on the Michigan–Wisconsin border. Shaded areas represent parameter 95% confidence intervals. In (a), Σ_20_PCB outlier to the far right removed for modeling

We examined the relationship between 26 five-year productivity means by river system and their corresponding *p,p’*-DDE and Σ_20_PCB geometric means. Neither the geometric mean of *p,p’*-DDE or of Σ_20_PCB was a significant predictor of productivity within five-year increments for each river reach (*p,p’*-DDE: *F*_1,24_ = 0.366, *p* = 0.551; Σ_20_PCB:* F*_1,23_ = 1.30 × 10^–4^,* p* = 0.991, Σ_20_PCB outlier removed for modeling; Fig. 3). Similarly, productivity models using observations from a broader set of territories (*n* = 819) including those where contaminants had not been measured also indicated that neither the geometric mean of *p,p’*-DDE or of Σ_20_PCB was a significant predictor of productivity within five-year increments for each river reach (*p,p’*-DDE: *p* = 0.857; Σ_20_PCB:* p* = 0.172, Σ_20_PCB outlier removed for modeling). However, productivity calculated only using territories with contaminant measurements likely overestimated productivity (only contaminant territories: 1.0–3.0 nestlings per nest, mean = 1.89, median = 1.85; all territories: 0.8–2.4 mean nestlings per nest, mean = 1.18, median = 1.10). Moreover, healthy productivity (≥ 1 nestling/nest) corresponded to mean Σ_20_PCB and *p,p’*-DDE levels in both datasets which would have been predicted to result in unhealthy productivity for this region in earlier time periods (Bowerman et al. 2003). Although the upper end of the range of Σ_20_PCB presented in Bowerman et al. (2003; 5–154 ng/g ww mean Σ_20_PCB) was not well represented in our study (only one point with a mean > 50 ng/g ww Σ_20_PCB, Fig. 3a), the ranges of mean *p,p’*-DDE were similar between studies (Bowerman et al. 2003: 3–35 ng/g ww *p,p’*-DDE; this study: 0.553–27.7 ng/g ww). This suggests that decreases in mean Σ_20_PCB but not mean *p,p’*-DDE could contribute to differences between the studies.

The results of our models suggest that, at concentrations measured during this study, Σ_20_PCB and *p,p’*-DDE did not have a strong effect on reproductive responses at the pre-fledgling stage in our study area. Model results also suggest that Σ_20_PCB and *p,p’*-DDE thresholds associated with healthy productivity in earlier time periods were no longer applicable during the time of this study as concentrations of multiple co-occurring contaminants have declined and other stressors, both chemical and ecological, may have changed as well. We show healthy productivity (≥ 1 eaglet per nest) at contaminant levels previously associated with unhealthy productivity for bald eagles in Michigan (healthy productivity thresholds at 35.4 ng/g ww Σ_20_PCB and 11.4 ng/g ww *p,p’*-DDE; Bowerman et al. 2003). These results also align with recent observations noting unexpectedly high productivity in bald eagles even with elevated *p,p’*-DDE and PCBs (Bush et al. 2020), further suggesting that these thresholds may no longer apply in the Great Lakes.

**Fig. 4:**
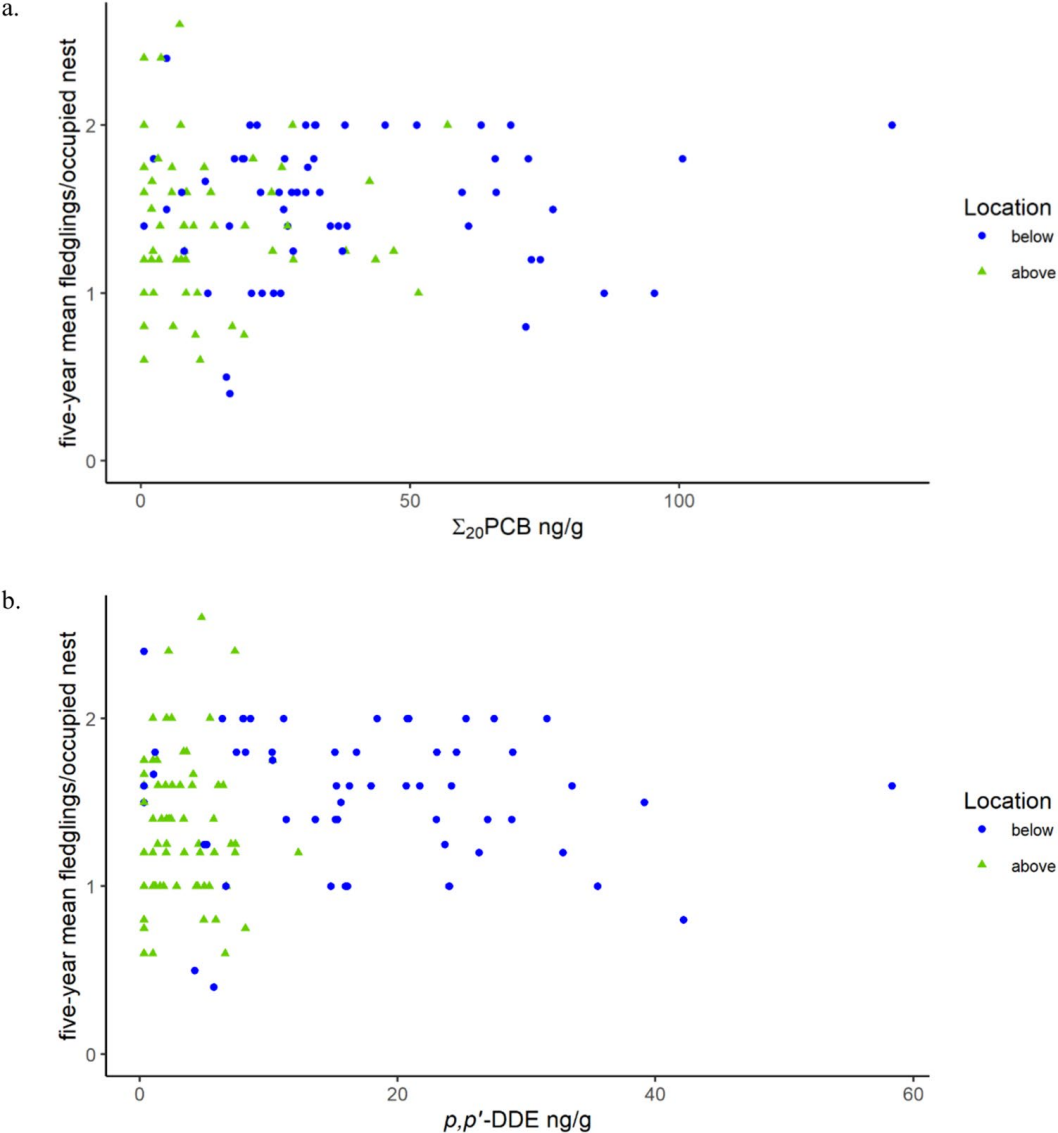
Relationship between **a** summed polychlorinated biphenyls (PCBs) and **b** *p,p’*-DDE in plasma of nestling bald eagles (*Haliaeetus leucocephalus*) at each territory during a single year and the associated five-year productivity means (nestlings/occupied nest) above and below lowermost dams along five river systems in the Lower Peninsula of Michigan and along the Michigan–Wisconsin border from 1999 to 2013

Our approach built upon that used by Bowerman et al. (2003, which included productivity estimate methods developed by Postupalsky 1974) but targeted smaller geographic areas and summarized contaminant data over a broader temporal scale (three vs. one five-year period). Recognizing these differences in approach, our methods produced slightly higher productivity estimates than methods used by Bowerman et al. (2003), yet our finding of a lack of a negative correlation between productivity and *p,p’*-DDE or Σ_20_PCB was also supported by examining individual territories (Table 3, Fig. 4). When examining mean nestlings per nest associated with a single contaminant sample by territory, of the 12 individual observations with < 1 nestling per nest (associated with unhealthy productivity), all except one had corresponding Σ_20_PCB values < 20 ng/g ww and *p,p’*-DDE values < 9 ng/g ww (71.4 ng/g ww Σ_20_PCB and 42.2 ng/g ww *p,p’*-DDE in the same observation; Fig. 4). Despite differences in approach, these results suggest a possible shift in how measured *p,p’*-DDE and PCBs alone relate to productivity in the Great Lakes in recent years and reflect the known challenge of determining thresholds of effects for individual factors in the field.

### Plasma Concentrations and Hazard Assessments

#### Σ_20_PCB

Concentrations of Σ_20_PCB were between 2.00 and 139 ng/g ww and exceeded the LOD in 81.1% of samples (107/132), with higher median concentrations below than above dams (Table 2, Fig. 2). These concentrations are similar or lower than those measured by others since the mid-1980s in nestling bald eagle plasma in areas of Michigan and the Great Lakes shorelines overlapping this study (Table 4).

**Table 4:** Concentrations of PCBs in bald eagle nestling plasma in areas comparable with this study from the mid-1980s

Study	Region	Comparable location (above, below)	Time	Central tendency (ng/g ww)	Range (ng/g ww)
This study	MI, WI—Lake Huron, Lake Michigan, interior Lower Peninsula of Michigan	Below	1999–2013	Median: 30.6, geo mean: 29.4	2.33–139
		above	1999–2013	Median: 8.47, geo mean: 9.90	2.00–57.0
Wierda et al. (2016)	MI—territories within 0.8 km of the lakes Michigan and Huron and their anadromous tributaries	Below	1999–2003	medians: 44 and 65	LOD-304
			2004–2008	medians: 34 and 40	LOD-141
	MI—inland territories in the upper and lower peninsulas > 0.8 km of the Great Lakes and not along anadromous tributaries	Above	1999–2003	medians: LOD and 4	LOD-189
			2004–2008	medians: 5 and 6	LOD-553
Venier et al. (2010)	MI—Territories near anadromous tributaries of lakes Michigan and Huron	Below	2005	mean: 76.2, median: 33.4	31.2–164
	MI—Inland territories nearest lakes Michigan and Huron	Above	2005	mean: 24.6, median: 15.5	5.46–52.8
Bowerman et al. (2003)	MI, WI—areas accessible to anadromous fish near Lake Michigan and Lake Huron shores	Below	1987–1992	geo means: 105–154	5–928
	MI—interior lower peninsula	Above	1987–1992	geo mean: 31	< 10–200
Datema (2012)	MI—territories > 8 km from Great Lakes and rivers accessible to anadromous fish	Above	1999–2003	mean: 7.0	–
			2004–2008	mean: 2.8	–
	MI—Territories within 8 km of the Great Lakes and rivers accessible to anadromous fish	Below	1999–2003	mean: 40.3	–
			2004–2008	mean: 44.6	–

Region describes the portion of a study area used for comparison. Comparable location notes if studied territories are most similar to above dam territories (food primarily sourced from inland) or below dam territories (food primarily sourced from the Great Lakes or Great Lakes tributaries accessible to anadromous fish).

When applying the nestling plasma TV associated with healthy productivity (35.4 ng/g ww, five-year means) suggested by Bowerman et al. (2003), samples with HQ > 1 were more common below lower most dams than above. However, we observed healthy mean productivity (≥ 1 nestling/nest) corresponding to all mean Σ_20_PCB values by river reach (five-year mean range: 0.558–100 ng/g ww; Fig. 3). Our examinations of mean nestlings per nest within individual territories provide further evidence that there was likely little influence of Σ_20_PCB on productivity within this study (see reproductive success modeling results and Fig. 4). Moreover, higher concentrations of Σ_20_PCB than we observed contributed to the development of a TV of 35.4 ng/g ww (Table 4, max in Bowerman et al. 2003: mean of 200 ng/g ww; max in this study: 139 ng/g ww for an individual territory).

Our results suggest that based on the conditions present during the years of our study and in the studied region, a TV of 35.4 ng/g ww may be overprotective and that a less protective TV, similar to those in estimated in other regions, may be more appropriate to calculate an updated HQ. For example, a TV of 189 ng/g ww in bald eagle nestling plasma has been associated with ≥ 1 nestling per nest in Green Bay, Lake Michigan (Elliott and Harris 2001/2002; one-year measure), and a geometric mean of 130 ng/g ww ΣPCBs (9.9–326 ng/g ww, 42 congeners) was measured in bald eagle nestling plasma during a time where productivity > 1.0 nestling/active nest along Lake Erie (1990–1996; Donaldson et al. 1999). Using either of these less protective values as a TV would indicate a HQ > 1 for, at most, one individual territory (139 ng/g ww) and for none of the geometric mean plasma concentrations by river reaches (max = 100 ng/g ww). This suggests that Σ_20_PCB minimally impairs bald eagle productivity as defined by Postupalsky (1974) in our studied area; however, we do not fully understand how this measure of productivity translates into the current ability of these eagle populations to be self-sustaining.

#### DDTs

Concentrations of *p,p’*-DDE were between 1.00 and 58.3 ng/g ww and detected in 86.4% of samples (114/132; see Online Resource 5 in supplemental data for *p,p’*-DDE recovery in standard reference material and for concentrations measured by others in areas overlapping this study), and concentrations of *p,p’*-DDD were between 0.387 and 18.2 ng/g ww and detected in 76.9% of samples (20/26), with overall lower concentrations above than below dams for both DDT metabolites (Table 2, Fig. 2). In the samples analyzed for all DDT metabolites (Table 1) *p,p’*-DDE was dominant, contributing 50.5–100% (geometric mean = 83.3%, median = 84.5%) of the total DDTs. The detected *p,p’*-DDE values in this study were slightly lower than or similar to concentrations observed by others in bald eagle nestling plasma in Michigan and throughout the Great Lakes between 1987 and 2008 (Wierda et al. 2016; Venier et al. 2010; Bowerman et al. 2003; Online Resource 5 in supplemental data). Between 1990 and 1994, Donaldson et al. (1999) measured geometric means of 20–60 ng/g ww *p,p’*-DDD in bald eagle nestling plasma from along lakes Erie and Huron. Although *p,p’*-DDD has been estimated to be roughly three times as lethal as *p,p’*-DDE in bird brains (Blus 2011), *p,p’*-DDE likely had the dominant effect in this study based on relative exposure levels. Of the samples examined for both analytes, 80.8% of samples (21/26) had at least three times as much *p,p’*-DDE as *p,p’*-DDD, and all samples had more *p,p’*-DDE than *p,p’*-DDD.

Previous studies have suggested that bald eagle nestling plasma concentrations below TVs of 11.4 ng/g ww *p,p’*-DDE (Bowerman et al. 2003) are associated with healthy productivity (five-year mean of 1.0 nestlings per occupied nest), and below 28.1 ng/g ww (Bowerman et al. 2003) or 27.8 ng/g (Elliott and Harris 2001/2002) are associated with the level of productivity needed to maintain a stable population (0.7 nestlings per occupied nest). Using the TV for a healthy population and the more protective of the two TVs for a stable population (27.8 ng/g ww) resulted in a determination that concentrations of *p,p’*-DDE were greater than would be indicated for a healthy or stable population in 28.8% (38/132) and 6.82% (9/132) of all samples, respectively (healthy HQ = 0.088–5.1; stable HQ = 0.036–2.1), but this was largely driven by the samples from below dams. For samples below dams, 63.8% (37/58) exceeded the TV for healthy populations while only one sample (1/74) from above dams exceeded this TV. However, similar to Σ_20_PCB, we observed healthy productivity (≥ 1 nestling/nest) corresponding to all mean *p,p’*-DDE values by river reach (five-year mean range: 0.553–27.7 ng/g ww; Fig. 3). Moreover, we did not observe a negative association between *p,p’*-DDE and productivity (productivity modeling, Fig. 3) and observed productivity at a similar rate of nestlings per nest for individual territories with *p,p’*-DDE values exceeding TVs for healthy and stable populations (Fig. 4). These results suggest that although *p,p’*-DDE appears to have remained elevated in eaglets, especially below dams, a TV for productivity as measured by Postupalsky (1974) was not applicable within the range of *p,p’*-DDE concentrations measured during the years of this study and within the study area.

#### Σ_12_PBDE; Dieldrin; High-Frequency Chlordanes: *Alpha*-Chlordane, *Cis*-Nonachlor, *Trans*-Nonachlor, and Oxychlordane; 1,2,4,5-Tetrachlorobenzene; and DL-PCBs

Concentrations of Σ_12_PBDE were between 1.13 and 24.5 ng/g ww and exceeded the LOD in 92.3% of samples (24/26). Of the 12 PBDEs, concentrations above the LOD were measured for PBDE-47, -49, -99, -100, -153, and -154, with PBDE-47, accounting for 73.6% of Σ_12_PBDE in all samples. The second and third most abundant congeners were PBDE-99 and -100, accounting for 12.4 and 10.7% of Σ_12_PBDE and detected in 8 and 9 samples, respectively. Concentrations of dieldrin were between 0.474 and 1.98 ng/g ww and exceeded the LOD in 30.8% (8/26) of samples. A*lpha*-chlordane was detected in 25.0% of samples (6/24, 0.180–0.626 ng/g ww), *cis-*nonachlor in 32.1% (9/28, 0.212–1.39 ng/g ww), *trans-*nonachlor in 57.1% (16/28, 0.255–2.27 ng/g ww), and oxychlordane in 25.0% (6/24, 0.551–0.933 ng/g ww). These chlordanes were detected at relatively low concentrations, near LODs. Concentrations of 1,2,4,5-tetrachlorobenzene were detected in 79.2% of samples (19/24, 0.729–1.22 ng/g ww). Concentrations were only slightly above LODs (LODs: 0.151–0.490 ng/g ww; Table 2, Fig. 2). PCB congeners with dioxin-like toxicity were measurable in all samples analyzed, resulting in DL-PCBs ranging from 1.28 × 10^–5^ to 2.03 × 10^–2^ ng/g ww (median = 1.63 × 10^–3^ ng/g ww, mean = 3.61 × 10^–3^ ng/g ww) across all samples. There were strong positive correlations of DL-PCBs with Total PCBs and with Σ_20_PCB (Online Resource 7 in supplemental data).

Concentrations of most of the other analytes frequently measured above LODs in plasma samples also were generally greater below than above dams, except for 1,2,4,5-tetrachlorobenzene (Table 2, Fig. 2). Median concentrations of Σ_12_PBDE did not significantly differ by Location (post hoc Wilcoxon rank sum test, *W* = 97 *p* = 0.112). DL-PCBs differed by Location (Wilcoxon rank sum *W* = 117, *p* = 0.00830; below dam median = 4.55 × 10^–3^ ng/g ww; above dam median = 8.47 × 10^–5^ ng/g ww) but not Year (Kruskal–Wallis rank sum χ^2^ = 6.16, *df* = 4, *p* = 0.188).

Concentrations of Σ_12_PBDE, dieldrin, and high-frequency chlordanes were similar to or lower than those detected in plasma sampled from bald eagle nestlings across Michigan and adjacent Great Lakes as reported in other studies. Measured concentrations of these contaminants indicate minimal potential for effects on bald eagle reproductive and population-level outcomes within studied sites at the concentrations measured in this study (Online Resource 6 in supplemental data). Currently, there is a lack of plasma-based information that would be needed to assess if the concentrations of 1,2,4,5-tetrachlorobenzene and DL-PCBs in this study are biologically meaningful for bald eagles (Online Resource 6 in supplemental data).

### Composition of Contaminant Mixtures

**Fig. 5:**
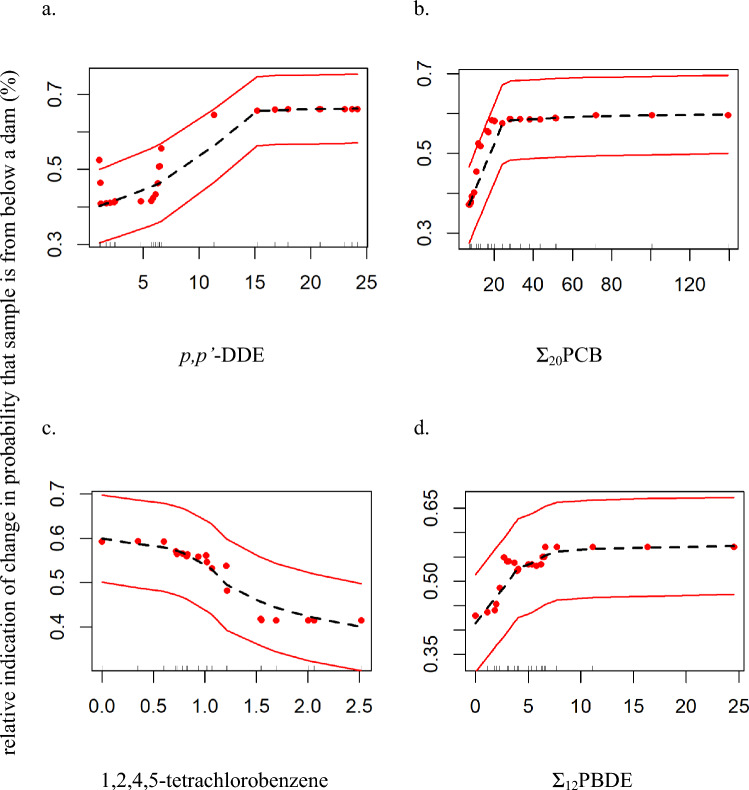
Partial dependence plots from Random Forest Analyses of the probability of occurring below the lowermost dam based on contaminant concentrations in bald eagle (*Haliaeetus leucocephalus*) nestling plasma from five river systems in Michigan, one of which is on the Michigan–Wisconsin border, 1999–2013. Responses (y-axis) are relative indications of changes in probability that nestling plasma is from below a dam. Predictors are *p,p’*-DDE (**a**), Σ_20_PCB (**b**), 1,2,4,5-tetrachlorobenzene (**c**), and Σ_12_PBDE (**d**). All units on x-axes are in ng/g ww. Dashed lines correspond to lowess smoothed lines representing the partial dependence between an explanatory variable and response. The solid lines indicate a smoothed error bar of ± two standard errors. The dots indicate the partial values used to fit the lowess function

The composition of contaminant mixtures differed by Location and Year when modeled using nonparametric MANOVA (Location: ½ LOD-substituted data set: *F*_1,23_ = 6.04, *R*^2^ = 0.192, *p* = 0.00400; zero-substituted data: *F*_1,23_ = 6.35, *R*^2^ = 0.224, *p* = 0.00400; Year: ½ LOD-substituted data set, only: F_1,23_ = 4.48, *R*^2^ = 0.142, *p* = 0.0340). For both ½ LOD-substituted and zero-substituted data, *p,p’*-DDE was the most important analyte discriminating between Locations above or below dam, followed by Σ_20_PCB (Online Resource 8 in supplemental data). 1,2,4,5-tetrachlorobenzene and Σ_12_PBDE were the next most valuable analytes for discriminating between Locations when using the zero-substituted data, but when using the ½ LOD-substituted data there was no notable difference between the usefulness of the remaining analytes after *p,p’*-DDE and Σ_20_PCB in discriminating between Locations (Online Resource 8 in supplemental data). Higher concentrations of *p,p’*-DDE, Σ_20_PCB, and Σ_12_PBDE and lower concentrations of 1,2,4,5-tetrachlorobenzene were more closely associated with downstream sample locations (Fig. 5). The contaminant concentrations were poor at discriminating among years (examined as a factor), with 70.83% out of bag estimated error rate (½ LOD), and thus contributions of individual contaminants were not examined. We measured lower overall *p,p’*-DDE and Σ_20_PCB concentrations than were measured in bald eagle plasma in the late 1980s and early 1990s at inland and Great Lakes territories comparable to above and below dam territories in Michigan (Bowerman et al. 2003). Despite these differences, these contaminant mixture results demonstrate that *p,p’*-DDE and PCBs continued to be useful indicators of contaminant mixtures in bald eagles through the time of this study. These contaminants also remain important in discriminating between above and below dam birds in the study area.

### Influence of Nest Location and Year on *p,p’*-DDE and Σ_20_PCB

We examined the influences of nest Location relative to the lowermost dam and Year (fixed effects) on *p,p’*-DDE and Σ_20_PCB using two model types: GLMM to account for a lack of independence in observations within River systems and Year (random effects) and regression by MLE for singly censored data (no random effects; only modeled influence on *p,p’*-DDE). *p,p’*-DDE concentrations above dams were approximately 21–28% of that below dams across both final models (½ LOD-substituted model: mean = 27.5%, 95% CI = 21.2–36.1%; non-substituted model: 21.0%, 20.4–21.6%; Tables 5 and 6). Σ_20_PCB concentrations in territories above dams were 19.8% of that below dams (95% CI 13.7–28.9%; Table 5). Percent differences were calculated using back transformation of coefficient estimates.

**Table 5:** Fixed effect coefficients with 95% confidence intervals and within-river system correlation (*r*) for models of natural log-transformed *p,p’*-DDE^a^ and Σ_20_PCB in bald eagle (*Haliaeetus leucocephalus*) nestling plasma above and below lowermost dams on five river systems in Michigan, one of which is on the Michigan–Wisconsin border, 1999–2013

Data	Response	*β*_0_	*β*_Location_	95% CI	*P*	*r*
half	ln(*p,p’*-DDE + 1)	2.67	− 1.29	− 1.55, − 1.02	< 0.001	0.296
half	ln(Σ_20_PCB + 1)	3.42	− 1.62	− 1.99, − 1.24	< 0.001	0.330

Location refers to position relative to a lower dam. The reference level for Location is below dams

^a^The *p,p’*-DDE concentrations reported for the 2014 samples analyzed through the Geochemical and Environmental Research Group (GERG) were not corrected for the low recovery of *p,p’*-DDE measured in the accompanying SRM

**Table 6:** Fixed effect coefficients with 95% confidence intervals for the model of natural log-transformed *p,p’*-DDE^a^ in bald eagle (*Haliaeetus leucocephalus*) nestling plasma using censored regression to account for values < LOD

Term	β	95% CI	*p*
intercept	2.41	2.38, − 2.43	< 0.001
Location	− 1.56	− 1.59, − 1.53	< 0.001
Year	− 0.0469	− 0.0503, − 0.0434	< 0.001

Data are from above and below lowermost dams on five river systems in Michigan, one of which is on the Michigan–Wisconsin border, 1999–2013. The reference level for Location is below dam. χ^2^ test was used to compare the final model to the full model (likelihood correlation coefficient (*r*), *df* = 6, 132, *p* < 0.001).

^a^The *p,p’*-DDE concentrations reported for the 2014 samples analyzed through the Geochemical and Environmental Research Group (GERG) were not corrected for the low recovery of *p,p’*-DDE measured in the accompanying SRM.

*p,p’*-DDE declined an average of 4.58% (95% CI 4.25–4.91%) annually based on the model using non-substituted data (Table 6). The *p,p’*-DDE decline is similar to the 3.0% annual decline in DDE concentration in bald eagle nestling plasma that Dykstra et al. (2010) detected from 1989 to 2008 in populations along Lake Superior. Similar annual declines could explain the decrease in *p,p’*-DDE in bald eagle nestling plasma between 1987 and 2008 in Michigan at inland and Great Lakes breeding areas (except for *p,p’*-DDE at Lake Huron breeding areas) observed by Wierda et al. (2016).

Year did not have an average fixed effect across all samples on Σ_20_PCB, which would have indicated an overall trend in changes in Σ_20_PCB concentrations over time. The lack of an annual decrease in Σ_20_PCB in bald eagle nestling plasma in this study contrasts with other examinations of bald eagle nestling plasma in the Great Lakes (4.0% annual PCB declines from 1987 to 2008, Dykstra et al. 2010; Wierda et al. 2016). Additionally, our measurements of Σ_20_PCB below and above dams were generally lower than in comparable inland and Great Lakes territories from the late 1980 to early 1990s presented in Bowerman et al. (2003). However, in recent years, rates of decline of PCBs have decreased across the Great Lakes and in Michigan. There were greater declines in PCBs in bald eagle nestling plasma between the 1987–1992 and 1999–2003 periods than between 1999–2003 and 2004–2008 periods (Wierda et al. 2016; Authors agreed to this retraction because they determined that some of the plasma samples run at Clemson University had failed quality assurance/quality control. In this retraction, it is noted that these samples were retested and concentrations were corrected and validated. A corrected version of this paper that includes the corrected and validated plasma concentrations is not available; however, we communicated with the authors to use the corrected and validated data in any of our comparisons for this paper.). PCB declines in herring gull eggs from colonies across the Great Lakes have generally slowed since the 1970s following an exponential decay model, with notably reduced declines since the early 2000s (de Solla et al. 2016). These rates of decline in herring gull eggs vary among locations and at some locations may have stabilized to the point that additional reductions are difficult to detect on a per year basis.

The final GLMMs of *p,p’*-DDE and Σ_20_PCB included random slopes and random intercepts, indicating that the relationship between Location and the modeled contaminant also varied by the combined random effects of River and Year (Table 7). Post hoc examination of random effects demonstrated differences among river systems, with Au Sable and Menominee having lower starting *p,p’*-DDE and Σ_20_PCB concentrations (lowest intercept values) and greater annual reductions (lowest slope values) than the other river systems (Table 7). Details on the influence of sample size by River and Location on Σ_20_PCB and *p,p’*-DDE concentrations is available in Online Resource 4 in supplemental data.

**Table 7:** Coefficients for intercept and slope by river system as influenced by random effects of River (intercept influence) and Year (slope influence) and estimated variance for River and Year across river systems where bald eagle (*Haliaeetus leucocephalus*) nestling plasma was sampled in Michigan and on the Michigan–Wisconsin border, 1999–2013

River/variance	*p,p’*-DDE^a^		Σ_20_PCB	
	*β*_r_	*β*_cyear_	*β*_r_	*β*_cyear_
Au Sable	− 0.888	− 0.107	− 0.885	− 0.0808
Menominee	− 0.807	− 0.0857	− 0.153	− 0.114
Manistee	0.179	− 0.0419	0.477	0.0252
Muskegon	0.238	− 0.00833	− 0.0861	0.0159
Saginaw	− 0.136	0.0304	0.394	0.0978
Variance	0.374	0.00521	0.575	0.0876

^a^The *p,p’*-DDE concentrations reported for the 2014 samples analyzed through the Geochemical and Environmental Research Group (GERG) were not corrected for the low recovery of *p,p’*-DDE measured in the accompanying SRM

It is possible that the actual concentrations of *p,p’*-DDE for a subset of samples processed by one laboratory in our dataset is greater than reported values and this would result in even greater significance of elevated *p,p’*-DDE concentrations in samples from below dams than is already indicated across all models examining the relationship between *p,p’*-DDE and Location. The 2014 samples analyzed through GERG were analyzed with an SRM for which the laboratory had low recovery of *p,p’*-DDE and were primarily samples from below dams (7/9 samples from below dam).

## Reconciliation of Established Threshold Values and Our Results

We conducted productivity analyses that were comparable with Bowerman et al. (2003) to understand if the productivity TV thresholds for Σ_20_PCB and *p,p’*-DDE developed using bald eagle measurements from 1987 to 1992 were applicable under the conditions present during our study for bald eagle breeding territories along tributaries of the Great Lakes. There have been conflicting results among studies examining the correlation of PCBs and *p,p’*-DDE with productivity in bald eagle (reviewed in Elliott and Harris 2001/2002). Despite these differences in results, the Bowerman et al. (2003) study most closely overlaps our study area and is currently used to assess impairments to bald eagle in the Great Lakes.

Bowerman et al. (2003) modeled strong negative associations of Σ_20_PCB and *p,p’*-DDE with productivity and, based on these models, provided TVs of 35.4 ng/g ww mean Σ_20_PCB and 11.4 ng/g ww mean *p,p’*-DDE associated with healthy productivity (≥ 1.0 nestlings per nest). Our work suggests that during the years of the study, these toxicity TVs alone were not predictors of bald eagle reproduction within our study area. We did not detect a negative relationship between Σ_20_PCB or *p,p’*-DDE and productivity, even when including nesting territories along rivers without contaminant measurements. We also observed healthy mean productivity for all river reaches when only using territories with contaminant measurements. Even when using observations from territories with and without contaminant measurements, 77% of river reaches had productivity of ≥ 0.8 nestlings per nest, which is supportive of a stable population (0.7 nestlings/occupied nest; Sprunt et al. 1973). Additionally, our analysis of contaminant mixtures shows that both Σ_20_PCB and *p,p’*-DDE are indicators of contaminant mixtures that can be used to differentiate between above and below dam territories (Fig. 5 and Online Resource 8 in supplemental data), which may have also been true in the late 1980s and early 1990s. Taken together, these results suggest that lower mean Σ_20_PCB and *p,p’*-DDE concentrations measured during the years of our study represent a reduction in toxicity of co-occurring contaminant mixtures relative to what was present from 1987 to 1992, when TV levels were derived. The toxicity of the full mixture of contaminants may have decreased to levels that, during our study period, were no longer sole predictors of productivity.

Previously established Σ_20_PCB and *p,p’*-DDE TVs may reflect past contaminant mixtures with higher toxicities than captured by this study. Reductions in overall mixture toxicity could influence the correlation between productivity and Σ_20_PCB or *p,p’*-DDE. During the period of our study, Σ_20_PCB and *p,p’*-DDE continued to be important indicators of overall contaminant mixtures in bald eagles and remained important in discriminating between above and below dam birds in the study. Updated Σ_20_PCB and *p,p’*-DDE TVs may continue to be useful as indicators of contaminant mixtures that are protective of bald eagle productivity. An updated TV for Σ_20_PCB is likely to be greater than the range of mean Σ_20_PCB values examined here, which were all associated with healthy productivity (Fig. 3; 0.558–40.5 ng/g ww, excluding one outlier at 100 ng/g). Other studies suggest higher ΣPCB concentrations may be associated with healthy bald eagle productivity (Elliott and Harris 2001/2002; Donaldson et al. 1999). Similar to Σ_20_PCB, an updated TV for *p,p’*-DDE is likely to be greater than the range of mean *p,p’*-DDE examined here (Fig. 3; 0.553–27.7 ng/g ww).

Food availability and nest turnover may also be factors in the differences in the relationships between productivity and Σ_20_PCB or *p,p’*-DDE found during our study period. Between the 1987–1992 and 1999–2013 periods, management of river flows changed and bald eagle populations throughout the Great Lakes increased. These changes resulted in shifts in population dynamics and more floating adults. Flow management changes affected the abundance and species composition of fish above and below lowermost dams (Rozich 1998; O’Neal 1997; Zorn and Sendek 2001; Schrouder et al. 2009) and thus also affected the contaminant mixtures in available food. Additionally, more floating adults reared in areas where food has relatively low contaminant levels (e.g., above dams) began to nest in areas where food had elevated contaminant levels (e.g., below dams), providing a short-term increased productivity in contaminated areas.

## Conclusion

The results from this study show that contaminant concentrations in bald eagle nestling plasma above lowermost dams are generally lower than below these dams across the five studied river systems in the Lower Peninsula of Michigan, one of which is on the Michigan–Wisconsin border. This demonstrates that dams impeding anadromous fish passage continue to reduce the movement of contaminants to bald eagle populations above dams. However, neither above nor below dam concentrations, in combination with the conditions at their respective locations during our study, were likely adversely affecting bald eagle productivity based on assessed endpoints. In general, reproductive success was greater below than above lowermost dams. This aligns with an overall shift in recent years toward no difference in, and in some cases greater, reproductive success in bald eagles near the Great Lakes compared to inland areas in Michigan (Wierda et al. 2010; Simon 2013; Bush et al. 2020).

We detected no negative correlation between productivity and Σ_20_PCB or *p,p’*-DDE (Table 3, Figs. 3 and 4). Moreover, productivity indicating a healthy population (≥ 1.0) was observed at concentrations of Σ_20_PCB and *p,p’*-DDE for which productivity < 1.0 would be expected based on past studies (Bowerman et al. 2003; Elliott and Harris 2001/2002). Because our results contrasted with the most recent literature, to examine the relationship between productivity and Σ_20_PCB and *p,p’*-DDE in bald eagle plasma in Michigan, we considered our modeling results along with the available toxicity values in the literature when conducting the hazard assessment. Our results suggest little risk to bald eagle reproduction from concentrations of dominant contaminants (Σ_20_PCB, Σ_12_PBDE, DDTs, dieldrin, *alpha*-chlordane, *cis*-nonachlor, *trans*-nonachlor, oxychlordane, and 1,2,4,5-tetrachlorobenzene) or DL-PCBs in our study area. Although a thorough hazard analysis would provide more robust risk assessment, this is currently impossible because little information is available about the toxicity of some measured contaminants or their influence on reproduction. Moreover, Σ_20_PCB and *p,p’*-DDE concentrations are indicative of differences in the complete measured contaminant mixtures above and below dams (Fig. 5 and Online Resource 8 in supplemental data). These relationships further suggest that overall contaminant mixtures, including contaminants not examined individually and possible additive and synergistic effects, did not hinder productivity in studied river reaches.

Our reassessment of the risk to bald eagle populations from dam removal and reestablishment of fish passage suggests risk has declined compared to the 1980s and 1990s. Because levels of contaminants below lowermost dams measured during our study did not appear to limit productivity measured at the pre-fledgling stage, removing barriers to anadromous fish passage could increase productivity of bald eagles at higher river reaches by increasing food availability. Similar to findings in Giesy et al. (1995), we found no reproductive impairment associated with *p,p’*-DDE concentrations. It is unlikely that during our study PCBs continued to represent the critical hazard in these river systems that was identified in bald eagle populations from 1989 to 1993 (Giesy et al. 1995). Additionally, source control efforts and natural attenuation appear to be reducing concentrations of at least some contaminants, as indicated by the model predicting *p,p’*-DDE concentrations (Table 6). At an approximate 4.5% annual reduction, the median and mean *p,p’*-DDE concentrations below dams would equal that measured for the above dam reaches in 11 years. Because *p,p’*-DDE was identified as an indicator of overall contaminant mixtures (Fig. 5 and Online Resource 8 in supplemental data), this may signal similar declines in other co-occurring contaminants.

Although dam removal at contaminant levels measured during our study is unlikely to reduce bald eagle reproductive success as indicated by nestling survival, dam removal may still increase risk to bald eagle populations via unmeasured endpoints. Additionally, measuring reproductive success at the pre-fledgling stage may fail to recognize effects of contaminants at juvenile and sub-adult stages, which may also affect population stability. For example, mortality associated with elevated plasma concentrations of dieldrin in great horned owl chicks can be delayed until months after fledge during dispersal (Frank and Lutz 1999). While not well understood in bald eagles, several endpoints may indicate reduced reproduction or population-level responses to contaminant levels throughout the Great Lakes. Endpoints such as altered immune response and biochemical and metabolic effects have been noted in other Great Lakes species (Grasman et al. in press; Grasman et al. 1996; Tseng, et al. 2022) suggesting further sublethal assessments maybe valuable. Additionally, research has shown bald eagles with relatively low contaminant levels dispersing from inland areas (i.e., floating adults) to breed along the Great Lakes may initially have greater breeding success than they would with each passing year as the parental contaminant burden increases (Kubiak et al. 1989; Peakall and Peakall 1973), thus creating artificially high productivity for short periods (Sénéchal et al. 2011). These conditions, coupled with greater adult mortality, may lead to the greater rates of nest turnover that have been documented near the Great Lakes compared to inland areas (Simon 2013). There is evidence that from the late 1970s to early 1990s these conditions created a population sink for bald eagles near the Great Lakes (Bowerman et al. 1995).

Examining the genetic structure of bald eagle populations could identify the prevalence of nest turnover and floating adults, which may be contributing to elevated productivity. Other stressors not considered here, such as emerging contaminants, food availability and quality, habitat quality, weather, and disease can impact reproductive success and have been identified as important for interpretation of the response of bald eagle population dynamics to contaminants (Elliott and Harris 2001/2002). We encourage future study of bald eagle population-level responses to contaminants concurrent with exposure to other stressors to better understand the complex and dynamic Great Lakes ecosystem.

The reduction in the risk to bald eagles from dam removal is a testament to the strides the Great Lakes region has taken over the past 50 years toward a cleaner environment that is healthier for the wildlife. During this time bald eagle populations have increased, and in 2007 the species was removed from the U.S. list of threatened and endangered species under the federal Endangered Species Act. The results presented here further describe a reduction in risk to bald eagle associated with reestablishing ecological connectivity between the Great Lakes and their tributaries. We suggest that at conditions present during the years of our study and in our study area, removing dams to establish connectivity may not pose excessive risk to bald eagle reproductive success from legacy contaminants. Our results also suggest that PCB and *p,p’*-DDE TVs derived in earlier time periods may not have been the best indicators of productivity in this system during the years of our study. This potential shift in our understanding of how contaminants may be influencing bald eagle populations throughout the Great Lakes highlights the need for additional information about their population dynamics. A better understanding of these population dynamics could provide a more nuanced picture of how bald eagles are recovering throughout the Great Lakes and to what degree these populations are self-sustaining. This information is also valuable to make informed decisions about how reestablishing fish passage on specific rivers may affect bald eagle reproductive success.

## Supplementary Information

Below is the link to the electronic supplementary material.

Supplementary file1 (DOCX 253 KB)
