# Supplementary material for: Examining Bald Eagle Contaminant Exposure and Reproductive Risk Above and Below Dams on Great Lakes Tributaries
Source: Arch Environ Contam Toxicol. 2024 Oct 17;87(4):353–74. doi: 10.1007/s00244-024-01090-w (PMC11585493; doi:10.1007/s00244-024-01090-w)
Supplement: Supplementary file 1 — Supplementary file1 (DOCX 253 KB) [file 244_2024_1090_MOESM1_ESM.docx]

**Title:** Examining Bald Eagle Contaminant Exposure and Reproductive Risk Above and Below Dams on Great Lakes Tributaries

**Journal Name:** Archives of Environmental Contamination and Toxicology

**Authors:** Carly J. Eakin*, Lisa L. Williams, Jeremy N. Moore, Mandy L. Annis, William W. Bowerman, David A. Best, Sarah Warner, Latice Fuentes, Kendall Simon, Brandon Armstrong

*corresponding author: U.S. Fish and Wildlife Service, [carly_eakin@fws.gov](mailto:carly_eakin@fws.gov)

# Electronic Supplementary Information

## Online Resource 1

#### Table SI-1. Analytical methods and reported units for the datasets used in the examination of bald eagle (*Haliaeetus leucocephalus*) nestling plasma above and below lowermost dams on five river systems in Michigan, one of which is on the Michigan-Wisconsin border, 1999-2013. Analyte-specific lipid removal and analysis methods are noted when they varied among analytes. GERG = Geochemical and Environmental Research Group, CIET = Clemson Institute of Environmental Toxicology, WSLH = Wisconsin State Lab of Hygiene; PCBs = polychlorinated biphenyls, OCs = organochlorines. All reported concentrations were converted to ng/g ww before being used.

| Lab | Years | Extraction (lipid removal and clean up) | Analyses | Reported units |
| --- | --- | --- | --- | --- |
| GERG | 2009-2013 | PCBs: solid-phase extraction (SPE), separatory funnel extraction (SFE), or continuous liquid/liquid extraction (CLLE); clean up with anthropogenic isolation column, gel-permeation chromatography (GPC), and/or silica gel or Florisil, as needed; purified by activated carbon and high-performance liquid chromatography (HPLC), as needed.  OCs and PBDEs: Extracted with addition of surrogate standards, Na_2_SO_4_, and methylene chloride in a centrifuge tube with a Teckmar Tissumizer; purified by silica/alumina column chromatography and HPLC, as needed. | OCs and PCBs: capillary gas chromatography with electron capture detector (CGC-ECD) and high resolution mass spectrometry (MS). PCBs following EPA Method 1668, Revision A (with corrections and changes through 8/20/03). OCs followed Wade et al., 1988.  PBDEs: CGC-ECD/MS with MS detector in the SIM mode; followed EPA 1614 protocols. | ppm |
| CIET | 1999-2008 | Details of extraction methods not reported in Wierda et al. 2016 | CGC-ECD, following EPA approved methods (Wierda et al. 2016). | ng/g |
| WSLH | 2002 | Lipids removed by GPC; clean up with silica gel or Florisil, as needed | OCs: CGC-ECD  PCBs: CGC-ECD | µg/L |
| WSLH | 2011-2013 | Lipids removed by GPC; clean up with silica gel or Florisil, as needed | CGC-ECD |  |

## Online Resource 2

#### Table SI-2. Generalized linear mixed model (GLMM) comparisons and Akaike Information Criterion values used to select optimal random model structures assessing (A) reproductive success based on individual nests (1997-2018), (B) reproductive success based on five-year means (1997-2018), and (C) chick plasma contaminant concentrations (1999-2013) for bald eagle (*Haliaeetus leucocephalus*) nestlings sampled in Michigan and on the Michigan-Wisconsin border. A full fixed effects model was used to determine optimal random model structure: no random effects, random intercept by River (1|River), or random slope and intercept by Year and River (1+Year|River). Fixed effects in models of reproductive success based on 5-year means only included Σ_20_PCB or *p,p’*-DDE because these contaminants were strongly correlated (*|r|* ≥ 0.7). Lowest AIC values indicate the best-fit models. ΔAIC denotes the difference between the best-fit models and other models.

| **Model** | **AIC** | **ΔAIC** |
| --- | --- | --- |
| **(A) Reproductive success – individual nests** |  |  |
| Model: Chicks per nest (CPN) |  |  |
| 1. CPN ~ Location * Year + (1+ Year\|River) | 2077.4 | 5.4 |
| 2. CPN ~ Location * Year + (1\|River) | 2073.7 | 1.7 |
| 3. CPN ~ Location * Year | 2072.0* |  |
| Model: Probability of nest success (PNS) |  |  |
| 1. PNS ~ Location * Year + (1+Year\|River) | 1014.5 | 70.28 |
| 2. PNS ~ Location * Year + (1\|River) | 1010.5 | 66.28 |
| 3. PNS ~ Location * Year | 944.22* |  |
| **(B) Reproductive success – five-year means** |  |  |
| Model: mean chicks per nest with Σ_20_PCB as a fixed effect (x̄ CPN_Σ20PCB_) |  |  |
| 1. x̄ CPN ~ Location * Year * Σ_20_PCB + (1 + Year\|River) | 203.87 | 5.71 |
| 2. x̄ CPN ~ Location * Year * Σ_20_PCB + (1\|River) | 200.16 | 2.0 |
| 3. x̄ CPN ~ Location * Year * Σ_20_PCB | 198.16* |  |
| Model: mean chicks per nest with *p,p’*-DDE as a fixed effect (x̄ CPN*_p,p’_*_-DDE_) |  |  |
| 1. x̄ CPN ~ Location * Year **p,p’*-DDE + (1+Year\|River) | ^a^ | - |
| 2. x̄ CPN ~ Location * Year * *p,p’*-DDE + (1\|River) | 194.13 | 2.0 |
| 3. x̄ CPN ~ Location * Year * *p,p’*-DDE | 192.13* |  |
| Model: mean percent nest success with Σ_20_PCB as a fixed effect (x̄ PNS_Σ20PCB_) |  |  |
| 1. x̄ PNS ~ Location * Year * Σ_20_PCB + (1 + Year\|River) | ^a^ | - |
| 2. x̄ PNS ~ Location * Year * Σ_20_PCB + (1\|River) | -43.259 | 2.0 |
| 3. x̄ PNS ~ Location * Year * Σ_20_PCB | -45.259* |  |
| Model: mean percent nest success with *p,p’*-DDE as a fixed effect (x̄ PNS*_p,p’_*_-DDE_) |  |  |
| 1. x̄ PNS ~ Location * Year * Σ_20_PCB * *p,p’*-DDE + (1+Year\|River) | ^a^ | - |
| 2. x̄ PNS ~ Location * Year * Σ_20_PCB * *p,p’*-DDE + (1\|River) | -53.613 | 2.0 |
| 3. x̄ PNS ~ Location * Year * Σ_20_PCB * *p,p’*-DDE | -55.613* |  |
| **(C) Chick plasma contaminant concentrations** |  |  |
| Model: Σ_20_PCB |  |  |
| 1. Σ_20_PCB ~ Location * Year + (1+Year\|River) | 391.78* |  |
| 2. Σ_20_PCB ~ Location * Year + (1\|River) | 400.03 | 8.25 |
| 3. Σ_20_PCB ~ Location * Year | 407.04 | 15.26 |
| Model: *p,p*’-DDE |  |  |
| 1. *p,p*’-DDE ~ Location * Year + (1+Year\|River) | 326.21 | 20.45 |
| 2. *p,p*’-DDE ~ Location * Year + (1\|River) | 308.86 | 3.1 |
| 3. *p,p*’-DDE ~ Location * Year | 305.76* |  |

^a^ The model failed to converge or had issues with singularity, therefore the random effect was dropped and this model was not considered

* Lowest AIC, indicating best-fit model

## Online Resource 3

#### Table SI-3. Summary statistics for contaminants in bald eagle (*Haliaeetus leucocephalus*) nestling plasma in river reaches above and below lowermost dams on five river systems in Michigan, one of which is on the Michigan-Wisconsin border, 1999-2013. Concentrations in ng/g ww. Min = minimum measured concentration, Max = maximum measured concentration, *sd* = standard deviation.

| Analyte | > DL (*n*) | *n* | River | Location | Median | Geo mean | Min | Max | *sd* |
| --- | --- | --- | --- | --- | --- | --- | --- | --- | --- |
| 1,2,3,4-tetrachlorobenzene | 0 | 1 | Au Sable | Above | - | - | - | - | - |
|  | 0 | 2 | Menominee | Below | - | - | - | - | - |
|  | 0 | 1 | Menominee | Above | - | - | - | - | - |
|  | 1 | 3 | Manistee | Below | 0.332 | - | - | - | - |
|  | 0 | 3 | Manistee | Above | - | - | - | - | - |
|  | 0 | 4 | Muskegon | Below | - | - | - | - | - |
|  | 0 | 4 | Muskegon | Above | - | - | - | - | - |
|  | 2 | 3 | Saginaw | Below | 1.45 | 1.11 | 0.509 | 2.40 | 1.34 |
|  | 0 | 3 | Saginaw | Above | - | - | - | - | - |
| 1,2,4,5-tetrachlorobenzene | 1 | 1 | Au Sable | Above | 1.69 | - | - | - | - |
|  | 2 | 2 | Menominee | Below | 1.11 | 1.11 | 1.01 | 1.21 | 0.140 |
|  | 1 | 1 | Menominee | Above | 0.822 | - | - | - | - |
|  | 3 | 3 | Manistee | Below | 0.602 | 0.547 | 0.349 | 0.780 | 0.217 |
|  | 3 | 3 | Manistee | Above | 1.07 | 1.27 | 0.937 | 2.06 | 0.615 |
|  | 2 | 4 | Muskegon | Below | 0.774 | 0.772 | 0.717 | 0.831 | 0.0806 |
|  | 3 | 4 | Muskegon | Above | 2.01 | 1.99 | 1.55 | 2.52 | 0.486 |
|  | 2 | 3 | Saginaw | Below | 1.12 | 1.11 | 1.02 | 1.22 | 0.140 |
|  | 2 | 3 | Saginaw | Above | 1.14 | 1.06 | 0.729 | 1.54 | 0.574 |
| aldrin | 0 | 1 | Au Sable | Above | - | - | - | - | - |
|  | 0 | 2 | Menominee | Below | - | - | - | - | - |
|  | 0 | 1 | Menominee | Above | - | - | - | - | - |
|  | 1 | 3 | Manistee | Below | 0.235 | - | - | - | - |
|  | 1 | 3 | Manistee | Above | 0.234 | - | - | - | - |
|  | 2 | 4 | Muskegon | Below | 0.265 | 0.254 | 0.191 | 0.338 | 0.104 |
|  | 0 | 4 | Muskegon | Above | - | - | - | - | - |
|  | 0 | 3 | Saginaw | Below | - | - | - | - | - |
|  | 0 | 3 | Saginaw | Above | - | - | - | - | - |
| *alpha*-BHC | 0 | 1 | Au Sable | Above | - | - | - | - | - |
|  | 1 | 2 | Menominee | Below | 0.197 | - | - | - | - |
|  | 0 | 1 | Menominee | Above | - | - | - | - | - |
|  | 0 | 3 | Manistee | Below | - | - | - | - | - |
|  | 1 | 3 | Manistee | Above | 0.271 | - | - | - | - |
|  | 0 | 4 | Muskegon | Below | - | - | - | - | - |
|  | 0 | 4 | Muskegon | Above | - | - | - | - | - |
|  | 0 | 3 | Saginaw | Below | - | - | - | - | - |
|  | 0 | 3 | Saginaw | Above | - | - | - | - | - |
| *alpha*-chlordane | 0 | 1 | Au Sable | Above | - | - | - | - | - |
|  | 0 | 2 | Menominee | Below | - | - | - | - | - |
|  | 0 | 1 | Menominee | Above | - | - | - | - | - |
|  | 1 | 3 | Manistee | Below | 0.274 | - | - | - | - |
|  | 1 | 3 | Manistee | Above | 0.180 | - | - | - | - |
|  | 1 | 4 | Muskegon | Below | 0.283 | - | - | - | - |
|  | 1 | 4 | Muskegon | Above | 1.15 | - | - | - | - |
|  | 2 | 3 | Saginaw | Below | 0.595 | 0.594 | 0.563 | 0.626 | 0.0446 |
|  | 0 | 3 | Saginaw | Above | - | - | - | - | - |
| chlorpyrifos | 1 | 1 | Au Sable | Above | 0.685 | - | - | - | - |
|  | 1 | 2 | Menominee | Below | 0.641 | - | - | - | - |
|  | 0 | 1 | Menominee | Above | - | - | - | - | - |
|  | 1 | 3 | Manistee | Below | 0.402 | - | - | - | - |
|  | 0 | 3 | Manistee | Above | - | - | - | - | - |
|  | 1 | 4 | Muskegon | Below | 0.415 | - | - | - | - |
|  | 0 | 4 | Muskegon | Above | - | - | - | - | - |
|  | 0 | 3 | Saginaw | Below | - | - | - | - | - |
|  | 1 | 3 | Saginaw | Above | 0.978 | - | - | - | - |
| *cis-*chlordane | 0 | 2 | Menominee | Below | - | - | - | - | - |
| *cis-*nonachlor | 0 | 1 | Au Sable | Above | - | - | - | - | - |
|  | 1 | 4 | Menominee | Below | 0.650 | - | - | - | - |
|  | 0 | 1 | Menominee | Above | - | - | - | - | - |
|  | 2 | 3 | Manistee | Below | 1.23 | 1.21 | 1.06 | 1.39 | 0.238 |
|  | 1 | 3 | Manistee | Above | 0.212 | - | - | - | - |
|  | 3 | 4 | Muskegon | Below | 0.772 | 0.673 | 0.474 | 0.833 | 0.192 |
|  | 0 | 4 | Muskegon | Above | - | - | - | - | - |
|  | 1 | 3 | Saginaw | Below | 0.922 | - | - | - | - |
|  | 1 | 3 | Saginaw | Above | 0.221 | - | - | - | - |
| *o,p’*-DDD | 0 | 1 | Au Sable | Above | - | - | - | - | - |
|  | 0 | 2 | Menominee | Below | - | - | - | - | - |
|  | 0 | 1 | Menominee | Above | - | - | - | - | - |
|  | 1 | 3 | Manistee | Below | 0.296 | - | - | - | - |
|  | 0 | 3 | Manistee | Above | - | - | - | - | - |
|  | 1 | 4 | Muskegon | Below | 0.174 | - | - | - | - |
|  | 0 | 4 | Muskegon | Above | - | - | - | - | - |
|  | 2 | 3 | Saginaw | Below | 1.11 | 0.941 | 0.526 | 1.69 | 0.820 |
|  | 0 | 3 | Saginaw | Above | - | - | - | - | - |
| *o,p’*-DDE | 0 | 1 | Au Sable | Above | - | - | - | - | - |
|  | 0 | 2 | Menominee | Below | - | - | - | - | - |
|  | 0 | 1 | Menominee | Above | - | - | - | - | - |
|  | 0 | 3 | Manistee | Below | - | - | - | - | - |
|  | 0 | 3 | Manistee | Above | - | - | - | - | - |
|  | 0 | 4 | Muskegon | Below | - | - | - | - | - |
|  | 0 | 4 | Muskegon | Above | - | - | - | - | - |
|  | 1 | 3 | Saginaw | Below | 0.908 | - | - | - | - |
|  | 0 | 3 | Saginaw | Above | - | - | - | - | - |
| *o,p’*-DDT | 0 | 1 | Au Sable | Above | - | - | - | - | - |
|  | 0 | 2 | Menominee | Below | - | - | - | - | - |
|  | 0 | 1 | Menominee | Above | - | - | - | - | - |
|  | 1 | 3 | Manistee | Below | 0.654 | - | - | - | - |
|  | 0 | 3 | Manistee | Above | - | - | - | - | - |
|  | 1 | 4 | Muskegon | Below | 0.287 | - | - | - | - |
|  | 0 | 4 | Muskegon | Above | - | - | - | - | - |
|  | 1 | 3 | Saginaw | Below | 1.25 | - | - | - | - |
|  | 0 | 3 | Saginaw | Above | - | - | - | - | - |
| *p,p’*-DDD | 1 | 1 | Au Sable | Above | 1.17 | - | - | - | - |
|  | 2 | 4 | Menominee | Below | 0.894 | 0.736 | 0.387 | 1.40 | 0.716 |
|  | 0 | 1 | Menominee | Above | - | - | - | - | - |
|  | 3 | 3 | Manistee | Below | 2.02 | 1.57 | 0.889 | 2.16 | 0.695 |
|  | 1 | 3 | Manistee | Above | 3.19 | - | - | - | - |
|  | 4 | 4 | Muskegon | Below | 1.42 | 1.56 | 0.748 | 4.16 | 1.54 |
|  | 3 | 4 | Muskegon | Above | 1.23 | 1.13 | 0.557 | 2.11 | 0.777 |
|  | 3 | 3 | Saginaw | Below | 11.3 | 10.0 | 4.92 | 18.2 | 6.65 |
|  | 3 | 3 | Saginaw | Above | 1.59 | 1.78 | 1.51 | 2.37 | 0.474 |
| *p,p’*-DDE | 5 | 6 | Au Sable | Below | 15.1 | 13.9 | 8.59 | 25.3 | 6.52 |
|  | 24 | 32 | Au Sable | Above | 2.80 | 2.88 | 1.00 | 8.19 | 2.21 |
|  | 5 | 5 | Menominee | Below | 2.50 | 3.30 | 1.07 | 24.0 | 9.77 |
|  | 16 | 20 | Menominee | Above | 2.15 | 2.37 | 1.00 | 10.3 | 2.70 |
|  | 13 | 13 | Manistee | Below | 24.6 | 22.7 | 6.39 | 58.3 | 14.3 |
|  | 7 | 7 | Manistee | Above | 4.00 | 3.97 | 1.20 | 12.3 | 3.72 |
|  | 16 | 16 | Muskegon | Below | 23.8 | 20.9 | 7.97 | 35.5 | 8.13 |
|  | 6 | 8 | Muskegon | Above | 5.80 | 5.17 | 2.49 | 7.07 | 1.61 |
|  | 16 | 18 | Saginaw | Below | 13.0 | 11.1 | 4.25 | 23.0 | 5.88 |
|  | 6 | 7 | Saginaw | Above | 3.95 | 3.71 | 1.85 | 6.30 | 1.86 |
| *p,p’*-DDT | 0 | 1 | Au Sable | Above | - | - | - | - | - |
|  | 0 | 4 | Menominee | Below | - | - | - | - | - |
|  | 0 | 1 | Menominee | Above | - | - | - | - | - |
|  | 1 | 3 | Manistee | Below | 0.218 | - | - | - | - |
|  | 0 | 3 | Manistee | Above | - | - | - | - | - |
|  | 0 | 4 | Muskegon | Below | - | - | - | - | - |
|  | 0 | 4 | Muskegon | Above | - | - | - | - | - |
|  | 1 | 3 | Saginaw | Below | 0.973 | - | - | - | - |
|  | 0 | 3 | Saginaw | Above | - | - | - | - | - |
| delta-BHC | 0 | 1 | Au Sable | Above | - | - | - | - | - |
|  | 0 | 2 | Menominee | Below | - | - | - | - | - |
|  | 0 | 1 | Menominee | Above | - | - | - | - | - |
|  | 0 | 3 | Manistee | Below | - | - | - | - | - |
|  | 0 | 3 | Manistee | Above | - | - | - | - | - |
|  | 0 | 4 | Muskegon | Below | - | - | - | - | - |
|  | 0 | 4 | Muskegon | Above | - | - | - | - | - |
|  | 0 | 3 | Saginaw | Below | - | - | - | - | - |
|  | 0 | 3 | Saginaw | Above | - | - | - | - | - |
| dieldrin | 0 | 1 | Au Sable | Above | - | - | - | - | - |
|  | 0 | 4 | Menominee | Below | - | - | - | - | - |
|  | 0 | 1 | Menominee | Above | - | - | - | - | - |
|  | 1 | 3 | Manistee | Below | 1.49 | - | - | - | - |
|  | 2 | 3 | Manistee | Above | 0.695 | 0.687 | 0.591 | 0.799 | 0.147 |
|  | 1 | 4 | Muskegon | Below | 1.05 | - | - | - | - |
|  | 1 | 4 | Muskegon | Above | 0.474 | - | - | - | - |
|  | 2 | 3 | Saginaw | Below | 1.51 | 1.44 | 1.06 | 1.97 | 0.644 |
|  | 1 | 3 | Saginaw | Above | 0.676 | - | - | - | - |
| endosulfan II | 0 | 1 | Au Sable | Above | - | - | - | - | - |
|  | 0 | 2 | Menominee | Below | - | - | - | - | - |
|  | 0 | 1 | Menominee | Above | - | - | - | - | - |
|  | 0 | 3 | Manistee | Below | - | - | - | - | - |
|  | 1 | 3 | Manistee | Above | 0.270 | - | - | - | - |
|  | 0 | 4 | Muskegon | Below | - | - | - | - | - |
|  | 0 | 4 | Muskegon | Above | - | - | - | - | - |
|  | 0 | 3 | Saginaw | Below | - | - | - | - | - |
|  | 0 | 3 | Saginaw | Above | - | - | - | - | - |
| endrin | 0 | 1 | Au Sable | Above | - | - | - | - | - |
|  | 0 | 2 | Menominee | Below | - | - | - | - | - |
|  | 0 | 1 | Menominee | Above | - | - | - | - | - |
|  | 1 | 3 | Manistee | Below | 0.211 | - | - | - | - |
|  | 0 | 3 | Manistee | Above | - | - | - | - | - |
|  | 1 | 4 | Muskegon | Below | 0.654 | - | - | - | - |
|  | 0 | 4 | Muskegon | Above | - | - | - | - | - |
|  | 0 | 3 | Saginaw | Below | - | - | - | - | - |
|  | 1 | 3 | Saginaw | Above | 0.526 | - | - | - | - |
| *gamma*-BHC | 0 | 1 | Au Sable | Above | - | - | - | - | - |
|  | 1 | 2 | Menominee | Below | 1.56 | - | - | - | - |
|  | 0 | 1 | Menominee | Above | - | - | - | - | - |
|  | 0 | 3 | Manistee | Below | - | - | - | - | - |
|  | 1 | 3 | Manistee | Above | 2.33 | - | - | - | - |
|  | 2 | 4 | Muskegon | Below | 0.850 | 0.758 | 0.465 | 1.23 | 0.544 |
|  | 0 | 4 | Muskegon | Above | - | - | - | - | - |
|  | 0 | 3 | Saginaw | Below | - | - | - | - | - |
|  | 1 | 3 | Saginaw | Above | 0.547 | - | - | - | - |
| *gamma*-chlordane | 0 | 1 | Au Sable | Above | - | - | - | - | - |
|  | 0 | 2 | Menominee | Below | - | - | - | - | - |
|  | 0 | 1 | Menominee | Above | - | - | - | - | - |
|  | 0 | 3 | Manistee | Below | - | - | - | - | - |
|  | 0 | 3 | Manistee | Above | - | - | - | - | - |
|  | 2 | 4 | Muskegon | Below | 0.184 | 0.184 | 0.174 | 0.194 | 0.0141 |
|  | 0 | 4 | Muskegon | Above | - | - | - | - | - |
|  | 0 | 3 | Saginaw | Below | - | - | - | - | - |
|  | 0 | 3 | Saginaw | Above | - | - | - | - | - |
| HCB | 0 | 1 | Au Sable | Above | - | - | - | - | - |
|  | 1 | 2 | Menominee | Below | 0.498 | - | - | - | - |
|  | 0 | 1 | Menominee | Above | - | - | - | - | - |
|  | 0 | 3 | Manistee | Below | - | - | - | - | - |
|  | 0 | 3 | Manistee | Above | - | - | - | - | - |
|  | 1 | 4 | Muskegon | Below | 0.179 | - | - | - | - |
|  | 0 | 4 | Muskegon | Above | - | - | - | - | - |
|  | 2 | 3 | Saginaw | Below | 0.827 | 0.812 | 0.670 | 0.983 | 0.221 |
|  | 0 | 3 | Saginaw | Above | - | - | - | - | - |
| heptachlor | 0 | 1 | Au Sable | Above | - | - | - | - | - |
|  | 0 | 2 | Menominee | Below | - | - | - | - | - |
|  | 0 | 1 | Menominee | Above | - | - | - | - | - |
|  | 0 | 3 | Manistee | Below | - | - | - | - | - |
|  | 0 | 3 | Manistee | Above | - | - | - | - | - |
|  | 1 | 4 | Muskegon | Below | 0.291 | - | - | - | - |
|  | 0 | 4 | Muskegon | Above | - | - | - | - | - |
|  | 0 | 3 | Saginaw | Below | - | - | - | - | - |
|  | 1 | 3 | Saginaw | Above | 0.394 | - | - | - | - |
| heptachlor epoxide | 0 | 1 | Au Sable | Above | - | - | - | - | - |
|  | 1 | 2 | Menominee | Below | 0.304 | - | - | - | - |
|  | 0 | 1 | Menominee | Above | - | - | - | - | - |
|  | 1 | 3 | Manistee | Below | 0.207 | - | - | - | - |
|  | 1 | 3 | Manistee | Above | 0.773 | - | - | - | - |
|  | 0 | 4 | Muskegon | Below | - | - | - | - | - |
|  | 1 | 4 | Muskegon | Above | 0.813 | - | - | - | - |
|  | 0 | 3 | Saginaw | Below | - | - | - | - | - |
|  | 1 | 3 | Saginaw | Above | 0.811 | - | - | - | - |
| mirex | 0 | 1 | Au Sable | Above | - | - | - | - | - |
|  | 0 | 2 | Menominee | Below | - | - | - | - | - |
|  | 0 | 1 | Menominee | Above | - | - | - | - | - |
|  | 0 | 3 | Manistee | Below | - | - | - | - | - |
|  | 1 | 3 | Manistee | Above | 0.258 | - | - | - | - |
|  | 1 | 4 | Muskegon | Below | 0.171 | - | - | - | - |
|  | 0 | 4 | Muskegon | Above | - | - | - | - | - |
|  | 0 | 3 | Saginaw | Below | - | - | - | - | - |
|  | 0 | 3 | Saginaw | Above | - | - | - | - | - |
| oxychlordane | 0 | 1 | Au Sable | Above | - | - | - | - | - |
|  | 0 | 2 | Menominee | Below | - | - | - | - | - |
|  | 0 | 1 | Menominee | Above | - | - | - | - | - |
|  | 1 | 3 | Manistee | Below | 0.363 | - | - | - | - |
|  | 1 | 3 | Manistee | Above | 0.551 | - | - | - | - |
|  | 3 | 4 | Muskegon | Below | 0.411 | 0.421 | 0.347 | 0.523 | 0.0891 |
|  | 0 | 4 | Muskegon | Above | - | - | - | - | - |
|  | 1 | 3 | Saginaw | Below | 0.933 | - | - | - | - |
|  | 0 | 3 | Saginaw | Above | - | - | - | - | - |
| Σ_20_PCB | 6 | 6 | Au Sable | Below | 32.3 | 26.9 | 4.78 | 65.7 | 20.6 |
|  | 16 | 32 | Au Sable | Above | 6.91 | 6.41 | 2.25 | 20.8 | 5.53 |
|  | 5 | 5 | Menominee | Below | 18.8 | 25.3 | 12.0 | 95.4 | 35.1 |
|  | 16 | 20 | Menominee | Above | 19.3 | 15.1 | 2.00 | 57.0 | 16.8 |
|  | 13 | 13 | Manistee | Below | 38.3 | 38.7 | 17.4 | 76.5 | 22.2 |
|  | 7 | 7 | Manistee | Above | 11.0 | 12.1 | 2.00 | 51.5 | 20.1 |
|  | 15 | 16 | Muskegon | Below | 27.3 | 27.3 | 7.54 | 74.2 | 16.8 |
|  | 6 | 8 | Muskegon | Above | 9.15 | 8.40 | 2.36 | 17.0 | 4.93 |
|  | 18 | 18 | Saginaw | Below | 30.8 | 27.5 | 2.33 | 139 | 37.8 |
|  | 5 | 7 | Saginaw | Above | 7.94 | 9.55 | 2.00 | 27.2 | 11.2 |
| pentachloroanisole | 0 | 1 | Au Sable | Above | - | - | - | - | - |
|  | 0 | 2 | Menominee | Below | - | - | - | - | - |
|  | 0 | 1 | Menominee | Above | - | - | - | - | - |
|  | 0 | 3 | Manistee | Below | - | - | - | - | - |
|  | 0 | 3 | Manistee | Above | - | - | - | - | - |
|  | 0 | 4 | Muskegon | Below | - | - | - | - | - |
|  | 0 | 4 | Muskegon | Above | - | - | - | - | - |
|  | 1 | 3 | Saginaw | Below | 0.452 | - | - | - | - |
|  | 0 | 3 | Saginaw | Above | - | - | - | - | - |
| TEQ_PCB_ | 1 | 1 | Au Sable | Above | 5.76 x 10^-5^ | - | - | - | - |
|  | 2 | 2 | Menominee | Below | 7.42 x 10^-4^ | - | 7.12 x 10^-5^ | 1.41 x 10^-3^ | 9.48 x 10^-4^ |
|  | 1 | 1 | Menominee | Above | 3.33 x 10^-5^ | - | - | - | - |
|  | 3 | 3 | Manistee | Below | 6.74 x 10^-3^ | - | 3.07 x 10^-3^ | 7.93 x 10^-3^ | 2.53 x 10^-3^ |
|  | 3 | 3 | Manistee | Above | 3.12 x 10^-3^ | - | 1.91 x 10^-4^ | 3.63 x 10^-3^ | 1.85 x 10^-3^ |
|  | 4 | 4 | Muskegon | Below | 1.02 x 10^-3^ | - | 9.44 x 10^-5^ | 6.04 x 10^-3^ | 2.79 x 10^-3^ |
|  | 4 | 4 | Muskegon | Above | 6.34 x 10^-5^ | - | 4.67 x 10^-5^ | 9.00 x 10^-5^ | 2.22 x 10^-5^ |
|  | 3 | 3 | Saginaw | Below | 1.90 x 10^-2^ | - | 7.21 x 10^-3^ | 2.03 x 10^-2^ | 7.22 x 10^-3^ |
|  | 3 | 3 | Saginaw | Above | 1.85 x 10-^-3^ | - | 1.28 x 10^-5^ | 3.61 x 10^-3^ | 1.80 x 10^-3^ |
| toxaphene | 0 | 1 | Au Sable | Above | - | - | - | - | - |
|  | 0 | 2 | Menominee | Below | - | - | - | - | - |
|  | 0 | 1 | Menominee | Above | - | - | - | - | - |
|  | 0 | 3 | Manistee | Below | - | - | - | - | - |
|  | 0 | 3 | Manistee | Above | - | - | - | - | - |
|  | 0 | 4 | Muskegon | Below | - | - | - | - | - |
|  | 0 | 4 | Muskegon | Above | - | - | - | - | - |
|  | 0 | 3 | Saginaw | Below | - | - | - | - | - |
|  | 0 | 3 | Saginaw | Above | - | - | - | - | - |
| *trans-*chlordane | 0 | 2 | Menominee | Below | - | - | - | - | - |
| *trans-*nonachlor | 0 | 1 | Au Sable | Above | - | - | - | - | - |
|  | 2 | 4 | Menominee | Below | 0.818 | 0.575 | 0.236 | 1.40 | 0.823 |
|  | 0 | 1 | Menominee | Above | - | - | - | - | - |
|  | 3 | 3 | Manistee | Below | 2.08 | 1.48 | 0.690 | 2.27 | 0.864 |
|  | 1 | 3 | Manistee | Above | 0.840 | - | - | - | - |
|  | 4 | 4 | Muskegon | Below | 1.62 | 1.44 | 0.871 | 1.90 | 0.476 |
|  | 2 | 4 | Muskegon | Above | 0.679 | 0.678 | 0.641 | 0.717 | 0.0537 |
|  | 2 | 3 | Saginaw | Below | 1.08 | 1.07 | 0.925 | 1.23 | 0.217 |
|  | 2 | 3 | Saginaw | Above | 0.822 | 0.595 | 0.255 | 1.39 | 0.801 |
| Σ_12_PBDE | 1 | 1 | Au Sable | Above | 5.76 | - | - | - | - |
|  | 4 | 4 | Menominee | Below | 2.91 | 2.71 | 1.51 | 4.22 | 1.12 |
|  | 1 | 1 | Menominee | Above | 3.93 | - | - | - | - |
|  | 3 | 3 | Manistee | Below | 6.37 | 5.37 | 3.67 | 6.61 | 1.63 |
|  | 2 | 3 | Manistee | Above | 1.56 | 1.50 | 1.13 | 1.98 | 0.601 |
|  | 4 | 4 | Muskegon | Below | 5.17 | 4.98 | 2.98 | 7.70 | 1.94 |
|  | 3 | 4 | Muskegon | Above | 4.06 | 3.93 | 2.30 | 6.50 | 2.11 |
|  | 3 | 3 | Saginaw | Below | 16.3 | 16.5 | 11.1 | 24.5 | 6.75 |
|  | 3 | 3 | Saginaw | Above | 3.99 | 3.56 | 1.82 | 6.21 | 2.20 |

## Online Resource 4

Sample size was positively correlated with the number of contaminants detected for a river system. Examining samples analyzed for > 2 analytes, the number of detected contaminants is moderately to strongly positively correlated with sample size (Pearson’s *r* = 0.883 pooled by River, *r* = 0.653 Locations represented separately, Table SI-3). Because of the correlation between detection and sample size, a lack of detection should not necessarily be assumed to indicate the absence of a contaminant in a river system or river reach.

Sample size may also influence detected contaminant concentrations, with more sample sizes associated with higher mean and median concentrations below dams and lower concentrations above dams. Sample size had a positive influence on median and geometric mean Σ_20_PCB and *p,p’*-DDE concentrations below dams (*r* = 0.258-0.629) and a negative influence on concentrations above dams (*r* = -0.737-0.0422). This supports the idea that dams prevent movement of these contaminants upstream.

#### Table SI-4. Number of contaminants detected in bald eagle (*Haliaeetus leucocephalus*) nestling plasma and samples analyzed by River and Location above or below lowermost dams on five river systems in Michigan, one of which is on the Michigan-Wisconsin border, 1999-2013.

| River | Contaminants detected > DL (above, below) | Samples analyzed for > 2 analytes (*p,p’*-DDE and Σ_20_PCBs) (above, below) | Total samples (above, below) |
| --- | --- | --- | --- |
| Au Sable | 6 (6, -) | 1 (1, -) | 38 (38, -) |
| Menominee | 12 (4, 12) | 5 (1, 4) | 25 (20, 5) |
| Manistee | 22 (16, 18) | 6 (3, 3) | 20 (7, 13) |
| Muskegon | 21 (9, 20) | 8 (4, 4) | 24 (8, 16) |
| Saginaw | 22 (13, 17) | 6 (3, 3) | 25 (7, 18) |

## Online Resource 5

#### Table SI-5. Concentrations of *p,p’*-DDE in bald eagle nestling plasma in referenced areas from the late-1980s to 2013. Region describes the portion of a study area used for comparison.

| Study | Region | Time | Central tendency (ng/g ww) | Range (ng/g ww) |
| --- | --- | --- | --- | --- |
| this study | MI, WI - Lake Huron, Lake Michigan, interior Lower Peninsula of Michigan | 1999-2013 | median: 6.42, geo mean: 6.50 | 1.00 and 58.3 |
| Venier et al. 2010 | MI - Territories near anadromous tributaries of lakes Michigan, Huron, and Superior as well as inland territories | 2005 | mean: 16.0, median: 17.3 | 2.85-41.7 |
| Wierda et al. 2016 | MI – territories in the upper and lower peninsulas along Great Lakes and inland | 1999-2008 | medians: 4-17 | DL-728 |
| Bowerman et al. 2003 | Michigan interior Lower Peninsula and along the Lake Michigan and Lake Huron shores | 1987-1992 | geo means: 10, 25, and 35, respectively | DL-235 |

Correcting for *p,p’*-DDE recovery in the standard reference material (SRM)

Although analysis of standard reference material (SRM) for samples analyzed by GERG in 2014 indicated that *p,p’*-DDE may be underrepresented in those samples given 30.8% and 36.8% recovery during that time period in the SRM, greater detection and concentrations of *p,p’*-DDE in these samples would be unlikely to alter the overall result of greater concentrations below than above dams. Most (7/9) of the samples analyzed in this batch of samples were below dams and had greater median *p,p’*-DDE than from above dams (below = 16.8 ng/g ww, above = 4.51 ng/g ww using zero-substituted data). Correcting for *p,p’*-DDE recovery in the SRM would, however, result in approximately three-fold greater concentration estimates for samples in that batch and thus a slightly greater percent of samples exceeding the TVs in the study area during times with stable and healthy levels of productivity (10.6% and 30.3%, respectively).

## Online Resource 6

### Comparisons of measured Σ_12_PBDE, dieldrin, high frequency chlordanes (alpha-chlordane, cis-nonachlor, trans-nonachlor, and oxychlordane), 1,2,4,5-tetrachlorobenzene, and TEQ_PCB_ with concentrations measured by others.

#### PBDEs

Above dam Σ_12_PBDE concentrations (1.13-6.50 ng/g ww) were similar to those measured by others in nestling bald eagles in Michigan from 2000-2012 (Venier et al. 2010; Guo et al. 2018) and in other areas of the northern Midwest from 2006-2011 (Route et al. 2014).

There is potential for reproductive effects of PBDEs in wild nestling bald eagles, but this relationship has not been established well enough to select a TV and calculate HQs in this study. Experimental studies have demonstrated adverse reproductive effects of PBDEs in some birds including European starling (*Sturnus vulgaris*) (Van den Steen et al. 2009) and American kestrel (*Falco sparverius*) (Fernie et al. 2009), and field observations have noted a decline in productivity associated with increased Σ_12_PBDE in osprey (*Pandion haliaetus*) (Henny et al. 2009). Although a review by Guigueno and Fernie (2017) suggests a general lack of effect of PBDEs on reproductive metrics in birds (e.g., Zebra finch, *Taeniopygia guttata* and *T. castanotis*; great skua, *Stercorarius skua*; glaucous gull, *Larus hyperboreus*), raptors appeared to be the most sensitive across the multiple endpoints examined, including reproduction, survival, and development. Additionally, liver concentrations of PBDEs measured in Michigan bald eagles were among the highest liver concentrations measured in wildlife worldwide, indicating Great Lakes bald eagles are exposed to a high level of PBDEs (Dornbos et al. 2015). Reproductive impairments associated with PBDEs have not been demonstrated for bald eagles; however, Venier et al. (2010) hypothesized that Σ_12_PBDE at levels they detected in nestling plasma (mean = 5.7 ng/g ww, median = 2.7) may contribute to poor reproduction observed in wild bald eagle populations in Michigan, with this hypothesis based on the concentrations of PBDEs in American kestrel eggs associated with reproductive impairments (Fernie et al. 2009) and assuming that bald eagle egg to plasma conversion factors for PBDEs may be similar to those for other OC pesticides and PCBs (Elliott and Harris 2001/2002). This runs contrary to observations of stable to increasing populations and healthy reproduction in areas with greater mean bald eagle nestling plasma concentrations of Σ_12_PBDE than measured in the Venier et al. (2010) study. Cesh et al. (2010) and McKinney et al. (2006) estimated mean plasma concentrations up to 30.9 ng/g ww in British Columbia and southern California, and Dykstra et al. (2005) calculated a geometric mean plasma concentration of 7.9 ng/g ww in Wisconsin along Lake Superior (Table SI-6).

Deleterious effects of PBDEs on thyroid hormones and vitamins needed for growth and development have been documented in birds including American kestrels (Fernie et al., 2006; Sullivan et al. 2010). Examining thyroid and vitamin responses could indicate effects of PBDEs during nestling development (i.e., during the same life stage at which productivity is assessed) with potential population level consequences. In bald eagle nestling plasma, no effect of 1.78-30.9 ng/g ww mean ∑_8_PBDEs on triiodothyronine (T3), thyroxine (T4), or retinol was detected by Cesh et al. (2010), further suggesting that Σ_12_PBDE concentrations within this range detected in this study have minimal potential for effects on bald eagle reproductive outcomes.

#### Table SI-6. Concentrations of PBDEs in bald eagle nestling plasma in referenced areas from the late-1980s to 2013. Region describes the portion of a study area used for comparison.

| Study | Region | Time | Central tendency (ng/g ww) | Range (ng/g ww) | PBDE Congeners |
| --- | --- | --- | --- | --- | --- |
| this study | MI, WI - Lake Huron, Lake Michigan, interior Lower Peninsula of Michigan | 1999-2013 | median: 4.14, geo mean: 4.42 | 1.13-24.5 | 12 PBDEs: 28, 47, 49, 66, 85, 99, 100, 138, 153, 154, 183, 209 |
| Venier et al. 2010 | MI - Territories near anadromous tributaries of lakes Michigan, Huron, and Superior as well as inland territories | 2005 | mean: 5.7, median: 2.7 | 0.35-29.3 | 40 congeners including all Σ_12_PBDE in this study |
| Guo et al. 2018 | MI – Territories near Great Lakes and rivers accessible to anadromous fish as well as inland territories | 2000-2012 | geo mean: 5.31 | 0.65-25.2 | ∑_35_PBDEs; the most abundant congeners were 47, 99, 100, 153, and 154 |
| Route et al. 2014 | northern Midwest | 2006-2011 |  | 1.78-12.0 | All congeners in this study except 49, 183, and 209 |
| Cesh et al. 2010 and McKinney et al. 2006 | British Columbia and southern California | 1998 and 2003 | means: 0.40-30.9 | DL-30.9 | 8 congeners included in this study: 47, 99, 100, 138, 153, 154, 183, and 209 |
| Dykstra et al. 2005 | Wisconsin along Lake Superior | 1989-2001 | mean: 8.4, geo mean: 7.9 | 6.1–13.6 | 9 congeners included in this study: 28, 47, 66, 85, 99, 100, 138, 153, and 154 |

#### Dieldrin

Overall, measured dieldrin concentrations in our study (between 0.474 and 1.98 ng/g ww) are lower than mean population concentrations in nestling bald eagle plasma sampled across Michigan adjacent to lakes Michigan and Huron in 2005 (concentrations > DL 2.76-4.97 ng/g ww; Venier et al. 2010). Our measured concentrations were also similar to those detected in nestling bald eagle plasma sampled in Michigan along the Great Lakes and in associated inland areas from 1999-2008 (five year means 0.00001-2.7 ng/g ww; Datema 2012), along the coast of British Columbia from 1993-1994 (> DL 0.1-0.8 ng/g ww; Elliott and Norstrom 1998), and in Minnesota, Wisconsin, and Michigan from 2006-2015 (DL-5.7 ng/g ww; Elliott et al. 2019).

Dieldrin concentrations measured by Elliott et al. (2019) and Venier et al. (2010) were from samples taken from reproductively stable to increasing bald eagle populations, but thresholds for dieldrin’s effects on reproductive performance have not been determined (reviewed by Elliott and Bishop 2011). Giesy et al. (1995) suggested that rather than examining the correlation of dieldrin concentrations with productivity, it may be more appropriate to consider the effects on other endpoints with population level consequences. Reduced post-fledgling survival has been associated with higher dieldrin concentrations in plasma concentrations in great horned owl (*Bubo virginianus*, > 100 ng/g ww compared with fledglings with < 50 ng/g ww concentrations; Frank and Lutz 1999). Based on their comprehensive review of effects of dieldrin on birds, Elliott and Bishop (2011) suggested a protective effect level for bird populations of 1,000 ng/g ww in adult plasma concentrations. Even if a safety factor of 10 were applied to account for potential differences in sensitives among species and between life stages (applied once to the endpoints associated with great horned owl post-fledglings in Frank and Lutz (1999) or applied twice to the effect level in Elliot and Bishop (2011)), the resulting TV of 5 or 10 ng/g ww would still be greater than the dieldrin concentrations measured in this study (HQ_TV5_ = 0.09-0.4, HQ_TV10_ = 0.05-0.20). These results suggest that it is unlikely that dieldrin is limiting for bald eagle populations within studied sites at the concentrations measured in this study.

#### High frequency chlordanes (alpha-chlordane, cis-nonachlor, trans-nonachlor, and oxychlordane)

*Alpha*-chlordane concentrations measured in this study (0.180-0.626 ng/g ww) were similar to or lower than those detected in plasma sampled from bald eagle nestlings across Michigan and adjacent Great Lakes in 2004 (1.0-3.6 ng/g ww; Wierda et al. 2008) and near lakes Michigan and Huron in 2005 (up to 10.0 ng/g ww; Venier et al. 2010). *Trans-*nonachlor, oxychlordane, and *cis-*nonachlor concentrations measured in this study (*trans-*nonachlor 0.255-2.27 ng/g ww, oxychlordane 0.551-0.933 ng/g ww, *cis-*nonachlor 0.212-1.39 ng/g ww) were within the range of the means measured in plasma sampled from bald eagle nestlings on the Pacific and Atlantic coasts. In British Columbia along the Pacific coast, *trans-*nonachlor was 0.5-2.5 ng/g ww (Cesh et al. 2008) and oxychlordane and *cis-*nonachlor concentrations were < 1.0 ng/g ww (Gill and Elliott 2003). In Newfoundland along the Atlantic coast, bald eagle nestling plasma was measured to have 0.8-3.9 ng/g ww *trans-*nonachlor, 0.2-3.6 ng/g ww oxychlordane, and DL-0.8 ng/g ww *cis-*nonachlor (Dominguez et al. 2003).

Concentrations of these chlordanes are below levels that likely would have a detectable effect on reproductive responses indicated by nestlings. For example, although Wierda et al. (2008) did not directly assess the effect of *alpha*-chlordane on bald eagle productivity, nestlings near the Great Lakes had lower concentrations of *alpha*-chlordane and lower productivity compared to nestlings on Great Lakes connecting channels with anadromous fish. This suggests that *alpha*-chlordane at these concentrations did not drive productivity. Additionally, *trans-*nonachlor and oxychlordane concentrations were similar to those in nestling bald eagle plasma along the coast of British Columbia, 1993-1994 (geometric means, *trans-*nonachlor: > DL 0.3-3 ng/g ww; oxychlordane: > DL 0.1-0.9 ng/g ww), where no relationship with chicks per nest was detected for either OC (Elliott and Norstrom 1998).

#### 1,2,4,5-tetrachlorobenzene

Concentrations of 1,2,4,5-tetrachlorobenzene (0.729-1.22 ng/g ww) were only slightly above DLs (DLs: 0.151-0.490 ng/g ww; Table 2, Fig. 2). 1,2,4,5-Tetrachlorobenzene concentrations in bald eagle plasma have not been reported elsewhere but have been reported in other studies that examined bird eggs and livers of reproductively healthy and reference populations of bald eagles and other birds. For example, 24-109 ng/g ww was measured in eggs of bald eagle in Alaska (pooled with hexachlorobenzene, Anthony et al. 2007), 10-63 ng/g ww was measured in liver of in Alaskan seabirds (Ricca et al. 2008), and geometric means of 3 and 7 ng/g ww were measured in barn swallow (*Hirundo rustica*) carcass and eggs, respectively (Custer et al. 2006). For comparison of relative plasma and egg concentrations of other chlorinated compounds, concentrations of *p,p’*-DDE and PCBs in bald eagle chick plasma are higher than corresponding concentrations in eggs and similar between chick plasma and eggs of great horned owls (*Bubo virginianus*) (Strause et al. 2007). PCB and *p,p’*-DDE plasma TVs are greater than egg TVs for bald eagle (Wiemeyer et al. 1993; Bowerman et al. 2003). Because conversions between species or tissues have not been confirmed for 1,2,4,5-tetrachlorobenzene in bald eagle, we cannot currently assess if differences in concentrations above and below dams indicate a biologically meaningful difference for eagles.

#### TEQ_PCB_

PCB congeners with dioxin-like toxicity were measurable in all samples analyzed, resulting in TEQ_PCB_ ranging from 1.28 x 10^-5^ to 2.03 x 10^-2^ ng/g ww (median = 1.63 x 10^-3^ ng/g ww, mean = 3.61 x 10^-3^ ng/g ww) across all samples. These values are similar to the PCB-based TEQs detected in bald eagle nestling blood along the Pacific Coast of Canada (approximately 3.0 x 10^-4^ to 1.4 x 10^-3^ ng/g ww), which were not significantly related to productivity (Elliott and Norstrom 1998). TEQ_PCB_ values in our study were also similar to the PCB and PCDD/PCDF-based TEQs detected in adult bald eagle blood in Michigan (2.0 x 10^-3^ to 1.2 x 10^-2^ ng/g; Kumar et al. 2002). Although conversion factors for TEQs among tissues and across species have not been established, these TEQ concentrations in plasma were well below the range of TCDD concentrations in eggs associated with critical thresholds for survival or reproduction in birds (summarized by Harris and Elliott 2011; lowest value is 0.18 ng/g ww in chicken egg). Similarly, bald eagle eggs (whole eggs) with a TEQ of 0.21 ng/g ww have been shown to not reduce hatching success (Elliott et al. 1996). An embryonic liver cell culture study indicated that bald eagles may be relatively insensitive to TCDD and related compounds compared to other species such as domestic chickens or ring-necked pheasants (Kennedy et al. 2003), with 0.10 ng/g ww in bald eagle yolk sacs suggested as a NOEL based on liver cell function (Elliott et al. 1996). However, TEQs measured in livers and kidneys in bald eagles in Michigan, up to 8.4 and 9.1 ng/g, respectively, exceeded toxicity thresholds for other avian species (Kumar et al. 2002). Currently there is a lack of plasma-based TEQ information that would be needed to assess if TEQ_PCB_ in this study influence reproductive success indicated by nestlings. It is possible that other unmeasured contaminants with dioxin-like toxicity, if included, could increase the total TEQ to be compared to such a threshold.

## Online Resource 7

Total PCBs and TEQ_PCB_ were strongly positively correlated (Pearson’s *r* = 0.911) as were Σ_20_PCB (calculated with ½ DL-substitution) and TEQ_PCB_ (*r* = 0.912), with both correlations being nearly identical (Fig. SI-1). Additionally, these correlations may indicate little difference in PCB mixtures and similar partitioning (i.e., depuration, uptake, and solubility rates) into plasma across sampled areas.
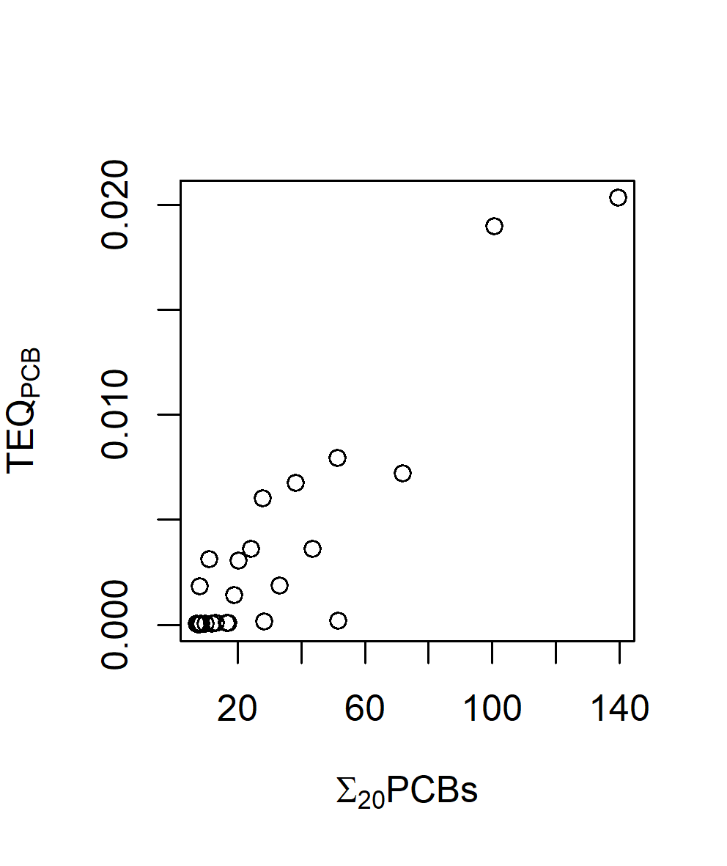


**Fig. SI-1.** Bald eagle (*Haliaeetus leucocephalus*) nestling plasma TEQ_PCB_ concentrations regressed against Σ_20_PCB (calculated with ½ DL-substitution; all units ng/g ww). A scatterplot of TEQ_PCB_ concentrations regressed against total PCBs was nearly identical to the figure shown here.

## Online Resource 8

| a. ½ DL-substituted | b. zero-substituted |
| --- | --- |
| 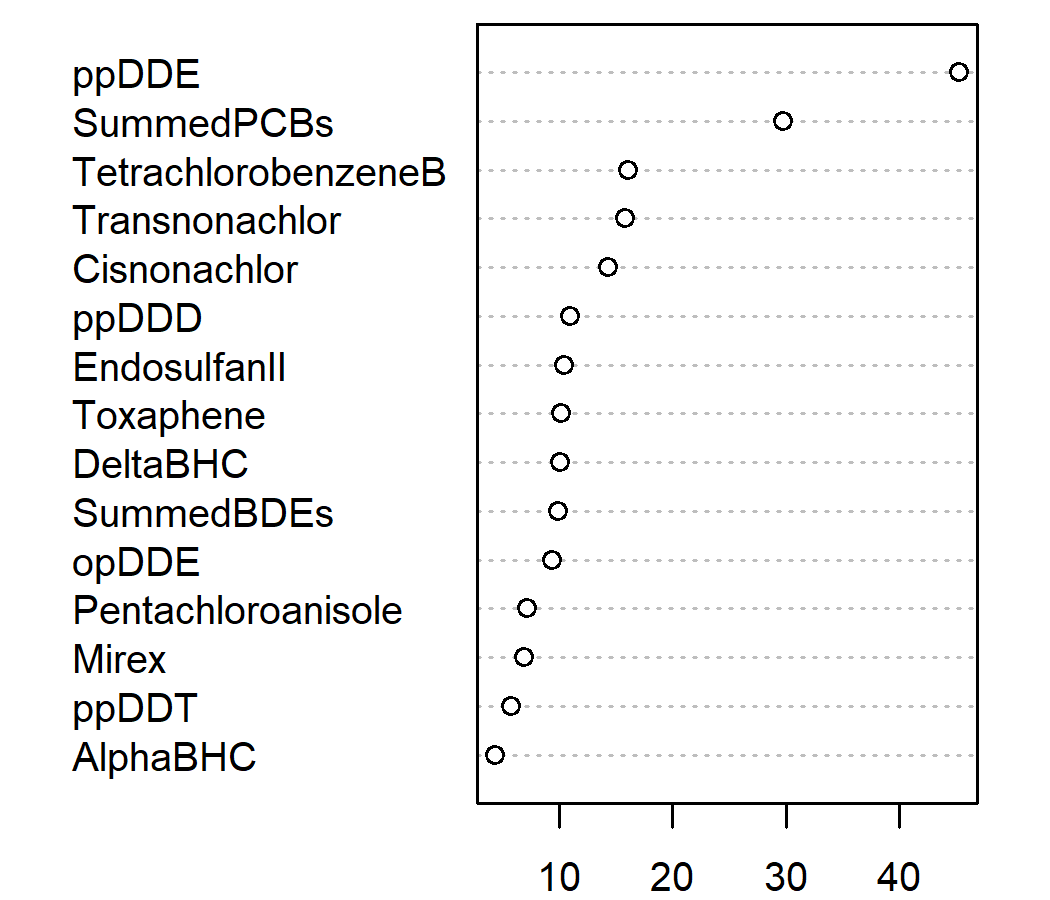  *p,p’-*DDE  Σ_20_PCB  1,2,4,5-tetrachlorobenzene  *trans-*nonachlor  *cis-*nonachlor  *p,p’-*DDD  endosulfan II  toxaphene  *delta*-BHC  Σ_12_PBDE  *o,p’-*DDE  pentachloroanisole  mirex  *p,p’-*DDT  *alpha*-BHC | 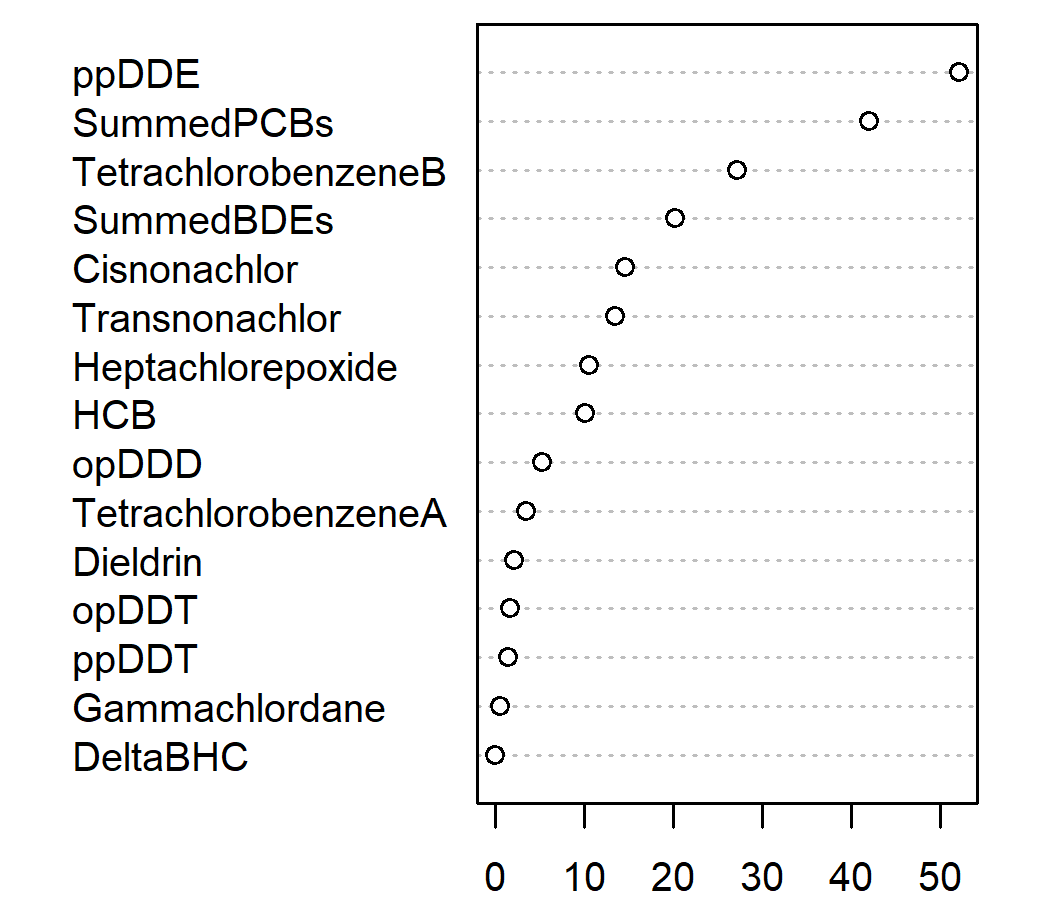  *p,p’-*DDE  Σ_20_PCB  1,2,4,5-tetrachlorobenzene  Σ_12_PBDE  *cis-*nonachlor  *trans-*nonachlor  heptachlor epoxide  HCB  *o,p’-*DDD  1,2,3,4-tetrachlorobenzene  dieldrin  *o,p’-*DDT  *p,p’-*DDT  *gamma*-chlordane  *delta*-BHC |
| Mean decrease in accuracy (%) | |

**Fig. SI-2.** Variable importance plots from classification trees of Location relative to a lowermost dam (above, below) based contaminant concentrations in bald eagle (*Haliaeetus leucocephalus*) nestling plasma sampled along five rivers systems in Michigan, one of which is on the Michigan-Wisconsin border, 1999-2013. Values ≤ DL were replaced with ½ DL (a) or zero (b). Plots show the rank-order of contaminants along the y-axis and the percent average decrease in mean square error when the values of the given variable are randomized while all others are held constant along the y-axis. Out of bag estimates of error rate was 20.8-25% for the ½ DL-substituted and 25% for the zero-substituted models.

# Literature Cited in Supplemental Information

Anthony RG, Miles AK, Ricca MA, Estes JA (2007) Environmental contaminants in bald eagle eggs from the Aleutian Archipelago. Environ Toxicol and Chem 26:1843-1855 https://doi.org/10.1897/06-334R.1

Bowerman WW, Best DA, Giesy JP, Shieldcastle MC, Meyer MW, Postupalsky S, Sikarskie JG (2003) Associations between regional differences in polychlorinated biphenyls and dichlorodiphenyldichloroethylene in blood of nestling bald eagles and reproductive productivity. Environ Toxicol and Chem 22:371-376 <https://doi.org/10.1002/etc.5620220218>

Cesh LS et al. (2010) Polyhalogenated aromatic hydrocarbons and metabolites: relation to circulating thyroid hormone and retinol in nestling bald eagles (*Haliaeetus leucocephalus*). Environ Toxicol Chem 29:1301-1310 https://doi.org/10.1002/etc.165

Cesh LS, Williams TD, Garcelon DK, Elliott JE (2008) Patterns and trends of chlorinated hydrocarbons in nestling bald eagle (*Haliaeetus leucocephalus*) plasma in British Columbia and Southern California. Arch Environ Contam Toxicol 55:496-502 https://doi.org/10.1007/s00244-007-9125-y

Custer TW, Custer CM, Goatcher BL, Melancon MJ, Matson CW, Bickham JW (2006) Contaminant exposure of barn swallows nesting on Bayou d'Inde, Calcasieu Estuary, Louisiana, USA. Environ Monit Assess 121:543-560 https://doi.org/10.1007/s10661-005-9153-x

Datema PP (2012) Using bald eagles to monitor hydroelectric projects license requirements along the Au Sable, Manistee, and Muskegon River, Michigan. Thesis, Clemson University

Dominguez L, Montevecchi WA, Burgess NM, Brazil J, Hobson KA (2003) Reproductive success, environmental contaminants, and trophic status of nesting bald eagles in eastern Newfoundland, Canada. J of Raptor Res 37:209-218

Dornbos P, Chernyak S, Rutkiewicz J, Cooley T, Strom S, Batterman S, Basu N (2015) Hepatic polybrominated diphenyl ether (PBDE) levels in Wisconsin river otters (*Lontra canadensis*) and Michigan bald eagles (*Haliaeetus leucocephalus*). J of Gt Lakes Res 41:222-227 https://doi: [10.1016/j.jglr.2014.12.023](https://dx.doi.org/10.1016%2Fj.jglr.2014.12.023)

Dykstra CR, Meyer MW, Rasmussen PW, Warnke DK (2005) Contaminant concentrations and reproductive rate of Lake Superior bald eagles, 1989–2001. J of Gt Lakes Res 31:227-235 https://doi.org/10.1016/S0380-1330(05)70253-7

Elliott JE, Bishop CA (2011) Cyclodiene and other organochlorine pesticides in birds. In: Beyer WN, Meador JP (eds) Environmental contaminants in biota. Second edn. CRC Press, Boca Raton, Florida, pp 447-476

Elliott JE, Harris ML (2001/2002) An ecotoxicological assessment of chlorinated hydrocarbon effects on bald eagle populations. Rev in Toxicol 4:1-60

Elliott JE, Norstrom RJ (1998) Chlorinated hydrocarbon contaminants and productivity of bald eagle populations on the Pacific coast of Canada. Environ Toxicol and Chem, 17:1142-1153 https://doi.org/10.1002/etc.5620170622

Elliott JE et al. (1996) Biological effects of polychlorinated dibenzo-*p*-dioxins, dibenzofurans, and biphenyls in bald eagle (*Haliaeetus leucocephalus*) chicks. Environ Sci and Technol 15:782-793 https://doi.org/10.1002/etc.5620150526

Elliott SM, Route WT, DeCicco LA, VanderMeulen DD, Corsi SR, Blackwell BR (2019) Contaminants in bald eagles of the upper Midwestern U.S.: a framework for prioritizing future research based on in-vitro bioassays. Environ Pollut 244:861-870 https://doi.org/10.1016/j.envpol.2018.10.093

Fernie KJ, Laird Shutt J, Ritchie IJ, Letcher RJ, Drouillard KG, Bird DM (2006) Changes in the growth, but not the survival, of American kestrels (*Falco sparverius*) exposed to environmentally relevant polybrominated diphenyl ethers. J of Toxicol and Environ Health 69:1541-1554 https://doi.org/10.1080/15287390500468753

Fernie KJ, Shutt JL, Letcher RJ, Ritchie IJ, Bird DM (2009) Environmentally relevant concentrations of DE-71 and HBCD alter eggshell thickness and reproductive success of American kestrels. Environ Sci and Technol 43:2124-2130 https://doi.org/10.1021/es8027346

Frank RA, Lutz RS (1999) Productivity and survival of great horned owls exposed to dieldrin. The Condor 101:331-339 https://doi.org/10.2307/1369996

Giesy JP et al. (1995) Contaminants in fishes from Great Lakes-influenced sections and above dams of three Michigan rivers. III. Implications for health of bald eagles. Arch of Environ Contamina and Toxicol 29:309-321 https://doi.org/10.1007/BF00212495

Gill CE, Elliott JE (2003) Influence of food supply and chlorinated hydrocarbon contaminants on breeding success of bald eagles. Ecotoxicol 12:95-111 https://doi.org/10.1023/a:1022549231826Grier JW, Elder JB, Gramlich FJ, Green NF, Kussman JV, Mathison JE, Mattson JP (1983) Northern states bald eagle recovery plan. U.S. Fish and Wildlife Service, Denver, Colorado

Guigueno MF, Fernie KJ (2017) Birds and flame retardants: a review of the toxic effects on birds of historical and novel flame retardants. Environ Res 154:398-424 https://doi.org/10.1016/j.envres.2016.12.033

Guo J, Simon K, Romanak K, Bowerman W, Venier M (2018) Accumulation of flame retardants in paired eggs and plasma of bald eagles. Environ Pollut 237:499-507 https://doi.org/10.1016/j.envpol.2018.02.056

Harris ML, Elliott JE (2011) Effects of polychlorinated biphenyls, dibenzo-*p*-dixoins and dibenzofurans, and polybrominated diphenyl ethers in wild birds. In: Beyer WN, Meador JP (eds) Environmental contaminants in biota. Second edn. CRC Press, Boca Raton, Florida, pp 477-530

Henny CJ, Kaiser JL, Grove RA, Johnson BL, Letcher RJ (2009) Polybrominated diphenyl ether flame retardants in eggs may reduce reproductive success of ospreys in Oregon and Washington, USA. Ecotoxicol 18:802-813 https://doi.org/10.1007/s10646-009-0323-4

Kennedy SW, Jones SP, Elliott JE (2003) Sensitivity of bald eagle (*Haliaeetus leucocephalus*) hepatocyte cultures to induction of cytochrome P4501A by 2,3,7,8-tetrachlorodibenzo-*p*-dioxin. Ecotoxicol 12:163-170 https://doi.org/10.1023/a:1022546509053

Kumar SK, Kannan K, Giesy JP, Masunaga S (2002) Distribution and elimination of polychlorinated dibenzo-*p*-dioxins, dibenzofurans, biphenyls, and *p,p‘*-DDE in tissues of bald eagles from the Upper Peninsula of Michigan. Environ Sci and Technol 36:2789-2796 <https://doi.org/10.1021/es0114660>

McKinney MA, Cesh LS, Elliott JE, Williams TD, Garcelon DK, Jetcher RJ (2006) Brominated flame retardants and halogenated phenolic compounds in North American west coast bald eaglet (*Haliaeetus leucocephalus*) plasma. Environ Sci and Technol 40:6275-6281 https://doi.org/10.1021/es061061l

Ricca MA, Keith Miles A, Anthony RG (2008) Sources of organochlorine contaminants and mercury in seabirds from the Aleutian archipelago of Alaska: inferences from spatial and trophic variation. Sci Total Environ 406:308-323 https://doi.org/10.1016/j.scitotenv.2008.06.030

Route WT, Dykstra CR, Rasmussen PW, Key RL, Meyer MW, Mathew J (2014) Patterns and trends in brominated flame retardants in bald eagle nestlings from the upper Midwestern United States.

Strause KD et al. (2007) Plasma to egg conversion factor for evaluating polychlorinated biphenyl and DDT exposures in great horned owls and bald eagles. Environ Toxicol and Chem 26:1399-1409 https://doi.org/10.1897/06-383r.1

Sullivan KM, Bird DM, Ritchie JI, Shutt JL, Letcher RJ, Fernie KJ (2010) Changes in plasma retinol of American kestrels (*Falco sparverius*) in response to dietary or in ovo exposure to environmentally relevant concentrations of a penta-brominated diphenyl ether mixture, DE-71. J Toxicol Environ Health A 73:1645-1654 https://doi.org/10.1080/15287394.2010.501720

Van den Steen E, Eens M, Covaci A, Dirtu AC, Jaspers VL, Neels H, Pinxten R (2009) An exposure study with polybrominated diphenyl ethers (PBDEs) in female European starlings (*Sturnus vulgaris*): toxicokinetics and reproductive effects. Environ Pollut 157:430-436 https://doi.org/10.1016/j.envpol.2008.09.031

Venier M, Wierda M, Bowerman WW, Hites RA (2010) Flame retardants and organochlorine pollutants in bald eagle plasma from the Great Lakes region. Chemosphere 80:1234-1240 https://doi.org/10.1016/j.chemosphere.2010.05.043

Wiemeyer SN, Bunck CM, Stafford CJ (1993) Environmental contaminants in bald eagle eggs - 1980-84 - and further interpretations of relationships to productivity and shell thickness. Arch of Environ Contamina and Toxicol. 24:213-227 https://doi.org/10.1007/BF01141351

Wierda MR, Leith KF, Parmentier K, Bowerman WW, Bush D, Sikarskie JG (2008) Michigan wildlife contaminant trend monitoring: year 2004 annual report nestling bald eagles, MI/DEQ/WB-08/085
